# Supplementary material for: The chromatin network helps prevent cancer-associated mutagenesis at transcription-replication conflicts
Source: Nat Commun. 2023 Oct 28;14:6890. doi: 10.1038/s41467-023-42653-0 (PMC10613258; doi:10.1038/s41467-023-42653-0)
Supplement: Supplementary file 1 — Supplementary Information [file 41467_2023_42653_MOESM1_ESM.pdf]

SUPPLEMENTARY INFORMATION

The chromatin network helps prevent cancer-associated mutagenesis at transcription-replication conflicts

Aleix Bayona-Feliu[1,2,3,*], Emilia Herrera-Moyano[1,2], Nibal Badra-Fajardo[1], Iván Galván-Femenía[3], María Eugenia Soler-Oliva[1,2] and Andrés Aguilera[1,2,*]

[1] *Centro Andaluz de Biología Molecular y Medicina Regenerativa CABIMER, Universidad de Sevilla-CSIC-Universidad Pablo de Olavide, Seville 41092, Spain*
[2] *Departamento de Genética, Facultad de Biología, Universidad de Sevilla, Seville 41012, Spain*
[3] *Institute for Research in Biomedicine (IRB Barcelona), The Barcelona Institute of Science and Technology (BIST), Barcelona, Spain*

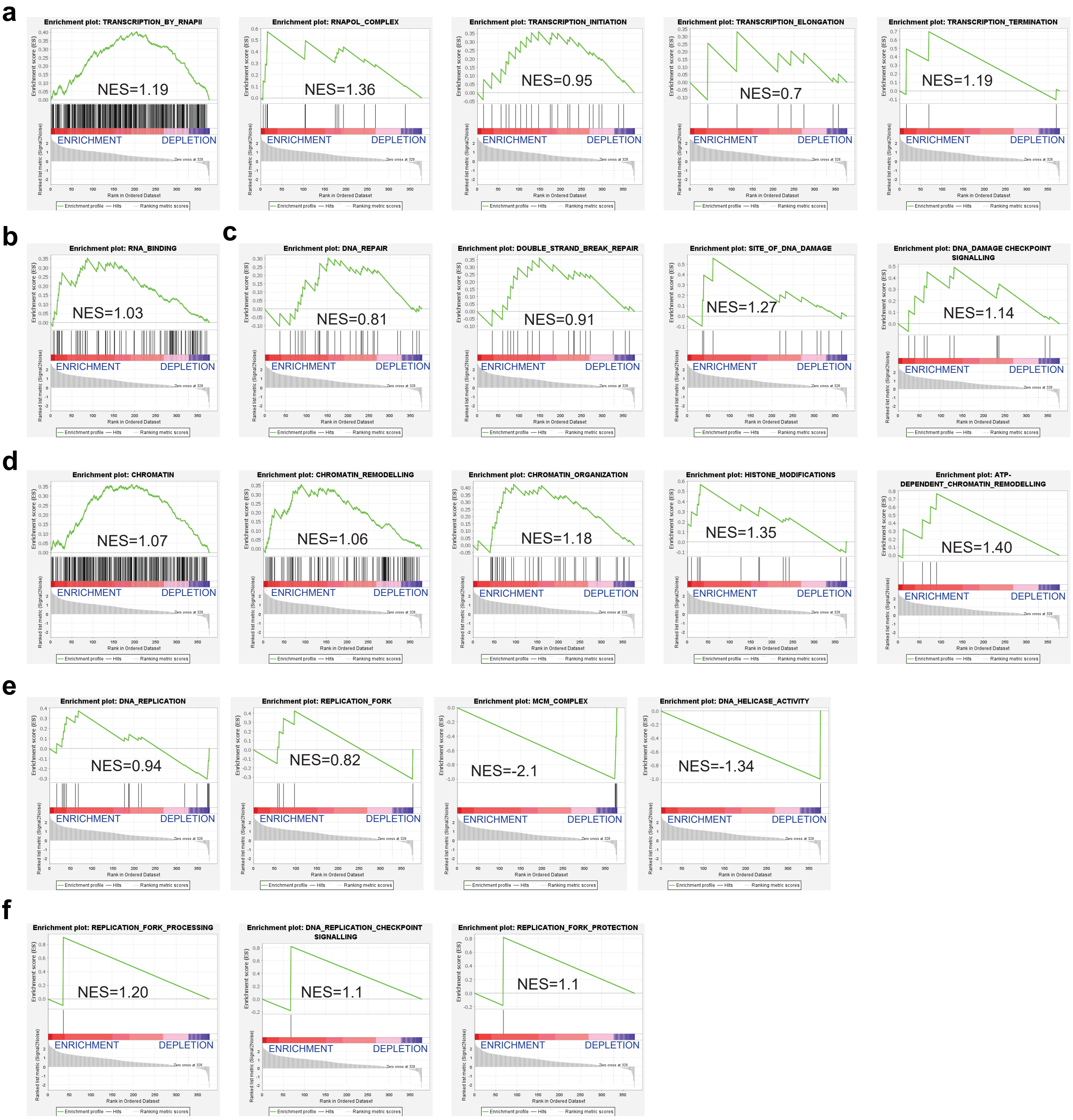

Supplementary Figure 1 – Factors enriched and depleted at transcription-replication conflicts (TRCs). a) Gene-set enrichment analysis (GSEA) of studied factors over transcription GO terms. **b)** Gene-set enrichment analysis (GSEA) of studied factors over RNA binding GO term. **c)** Gene-set enrichment analysis (GSEA) of studied factors over DNA damage GO terms. **d)** Gene-set enrichment analysis (GSEA) of studied factors over chromatin GO terms. **e)** Gene-set enrichment analysis (GSEA) of studied factors over DNA replication GO terms. **f)** Gene-set enrichment analysis (GSEA) of studied factors over replication fork stalling GO terms.

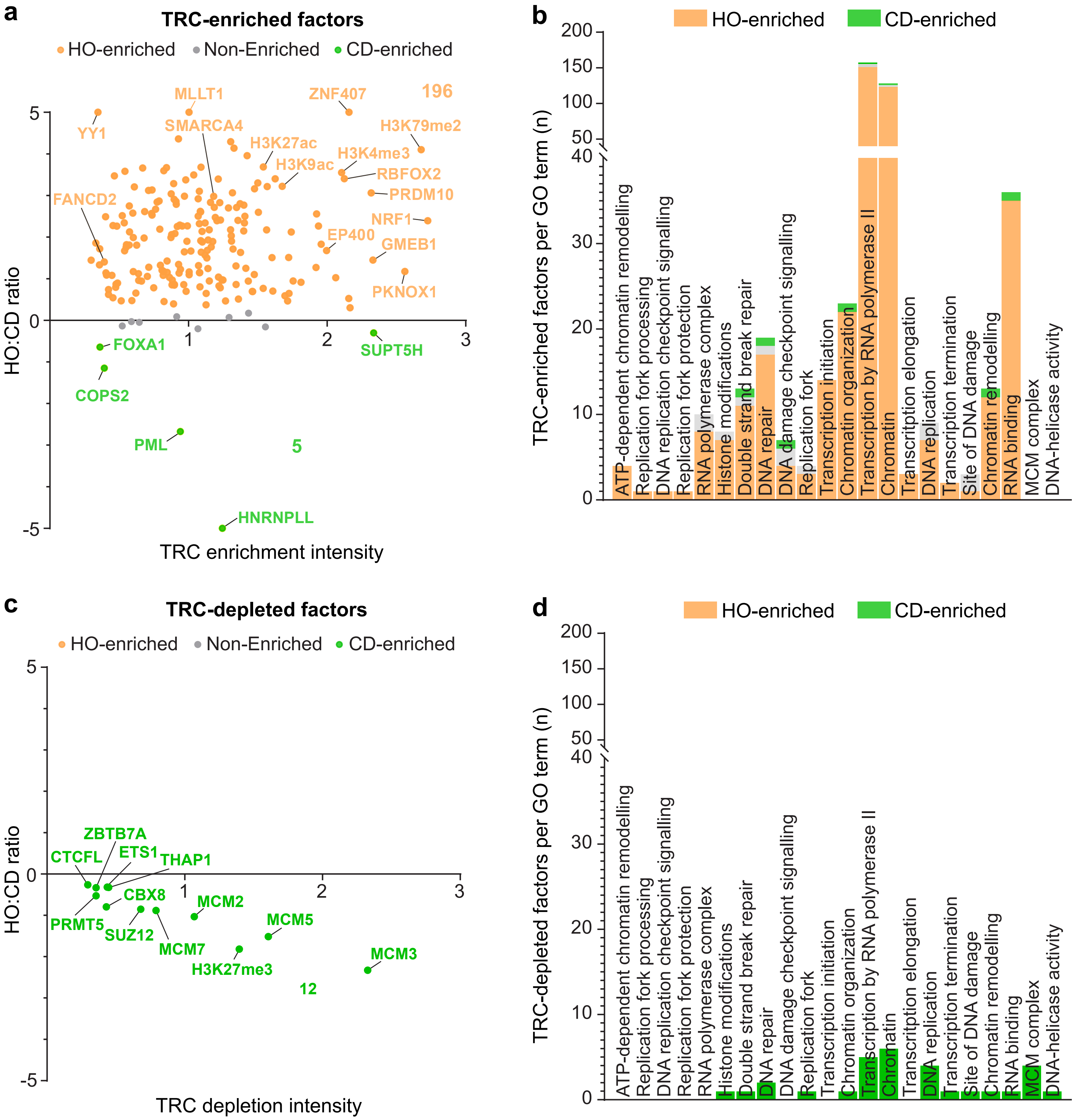

Supplementary Figure 2 – Factors enriched and depleted at transcription-replication conflicts (TRCs). a) Scatter plot showing the head-on:co-directional ratio of the TRC-enriched factors identified in the screening as function of the mean enrichment value. **b)** Number of TRC-enriched factors for each of the GO categories analyzed. **c)** Scatter plot showing the head-on:co-directional ratio of the TRC-depleted factors identified in the screening as function of the mean enrichment value. **d)** Number of TRC-depleted factors for each of the GO categories analyzed.
Source data are provided as a Source Data file. TRC: transcription-replication conflict; HO: head-on; CD: co-directional.

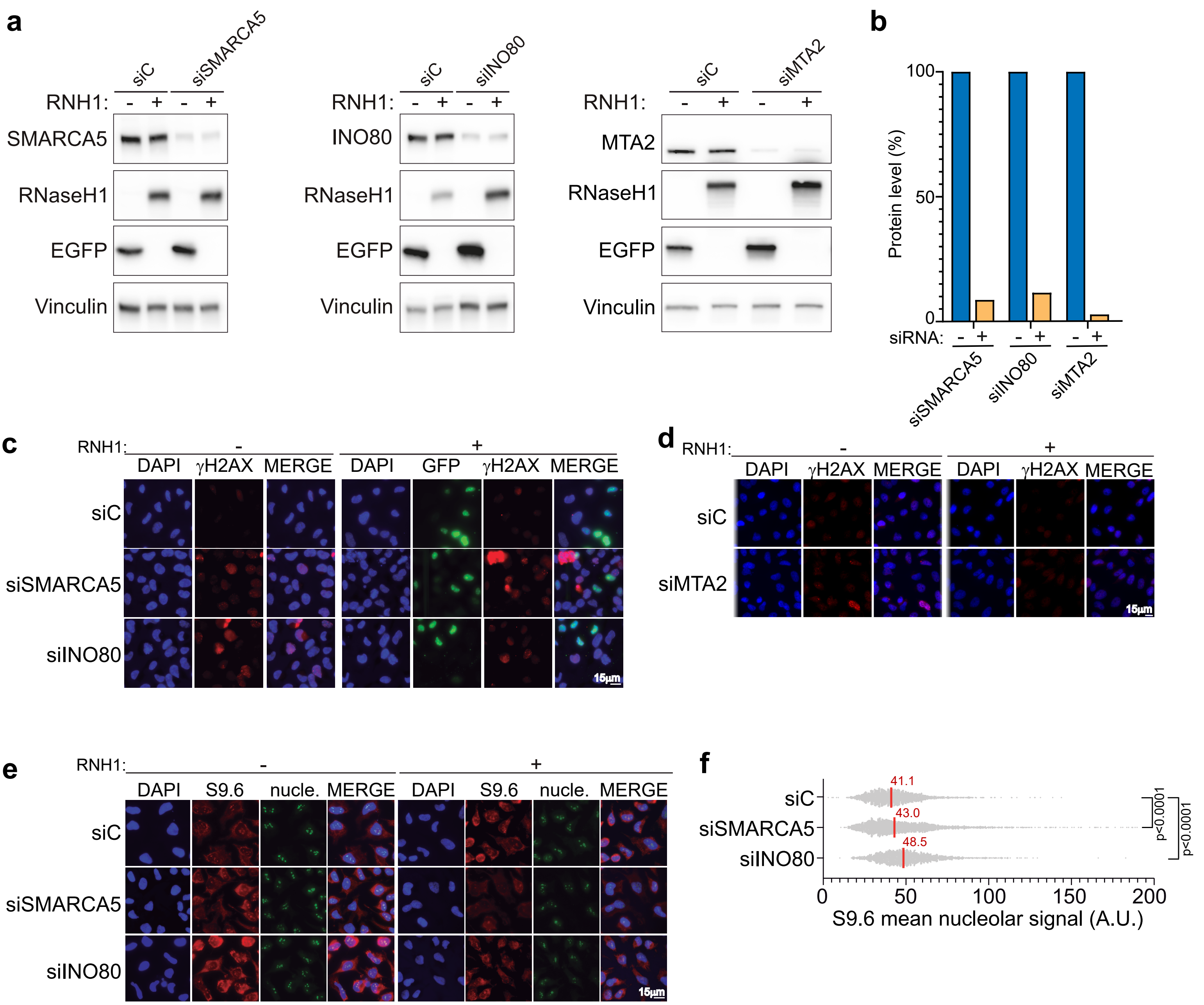

Supplementary Figure 3 – Assessment of DNA damage in siSMARCA5, siINO80, siMTA2 cells. a) Depletion controls by Western Blot. Immunoblotting examples against SMARCA5, INO80 and MTA2 showing effective protein depletion (left panels). EGFP and RNH1 antibodies used to check plasmid overexpression. Vinculin antibody used as loading control. **b)** Quantification of protein depletion as compared to control (siC) condiction. **c)** Example images of IF against γH2AX (red) in siC, siSMARCA5 and siINO80 cells overexpressing (+) or not (-) RNH1. GFP signal (green) used to detect RNH1 overexpression. **d)** Example images of IF against γH2AX (red) in siC and siMTA2 cells overexpressing (+) or not (-) RNH1. GFP signal (green) used to detect RNH1 overexpression. **e)** Example S9.6 (red) IFs images of siC, siSMARCA5 and siINO80 cells overexpressing (+) or not (-) RNH1. Nucleoli stained using nucleolin antibody (green). **f)** Quantification of nucleolar S9.6 mean signal intensity in the same conditions. Data presented as scatter plot (n=3). Median values are indicated. (Mann-Whitney U test, two-tailed).
Scale bars are indicated. Source data are provided as a Source Data file.

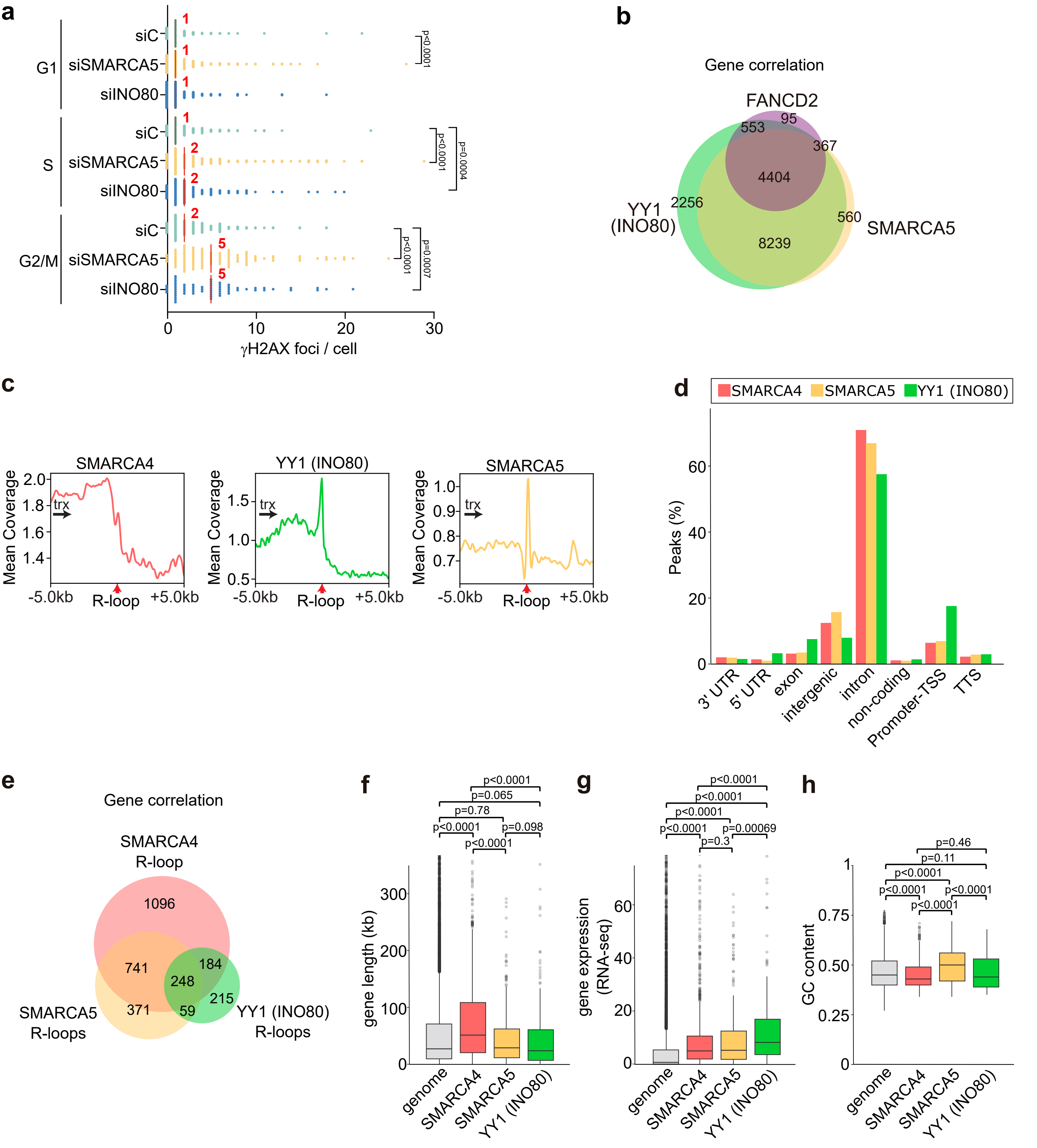

Supplementary Figure 4 – Analysis of DNA damage along the cell cycle in siINO80 and siSMARCA5 cells.
a) Quantification of γH2AX foci/cell in control (siC), siSMARCA5 and siINO80 cells along the cell cycle. Data presented as scatter plot (n=3). Median values are indicated. (Mann-Whitney U test, two-tailed). **b)** Venn diagram showing FANCD2 (purple), SMARCA5 (yellow) and INO80 (green) genome-wide co-occurrence in control K562 cells. **c)** Metapeak analysis of SMARCA4, SMARCA5 and YY1 across +/- 5kb R-loops sites. Transcription direction is indicated. **d)** Annotation of SMARCA4 (red), SMARCA5 (yellow) and YY1 (green) ChIP-seq peaks. **e)** Venn diagram showing correlation between R-loop-prone gene targets of SMARCA4 (red), SMARCA5 (yellow) and YY1 (green) in control K562 cells. **f)** Analysis of the length of R-loop-prone gene targets of SMARCA4 (red), SMARCA5 (yellow) and YY1 (green) in control K562 cells. (Wilcoxon test, two-tailed). **g)** Evaluation of the expression of R-loop-prone gene targets of SMARCA4 (red), SMARCA5 (yellow) and YY1 (green) in control K562 cells. (Wilcoxon test, two-tailed). **h)** Study of the GC content of R-loop-prone gene targets of SMARCA4 (red), SMARCA5 (yellow) and YY1 (green) in control K562 cells. (Wilcoxon test, two-tailed).
P-values are indicated. Source data are provided as a Source Data file.

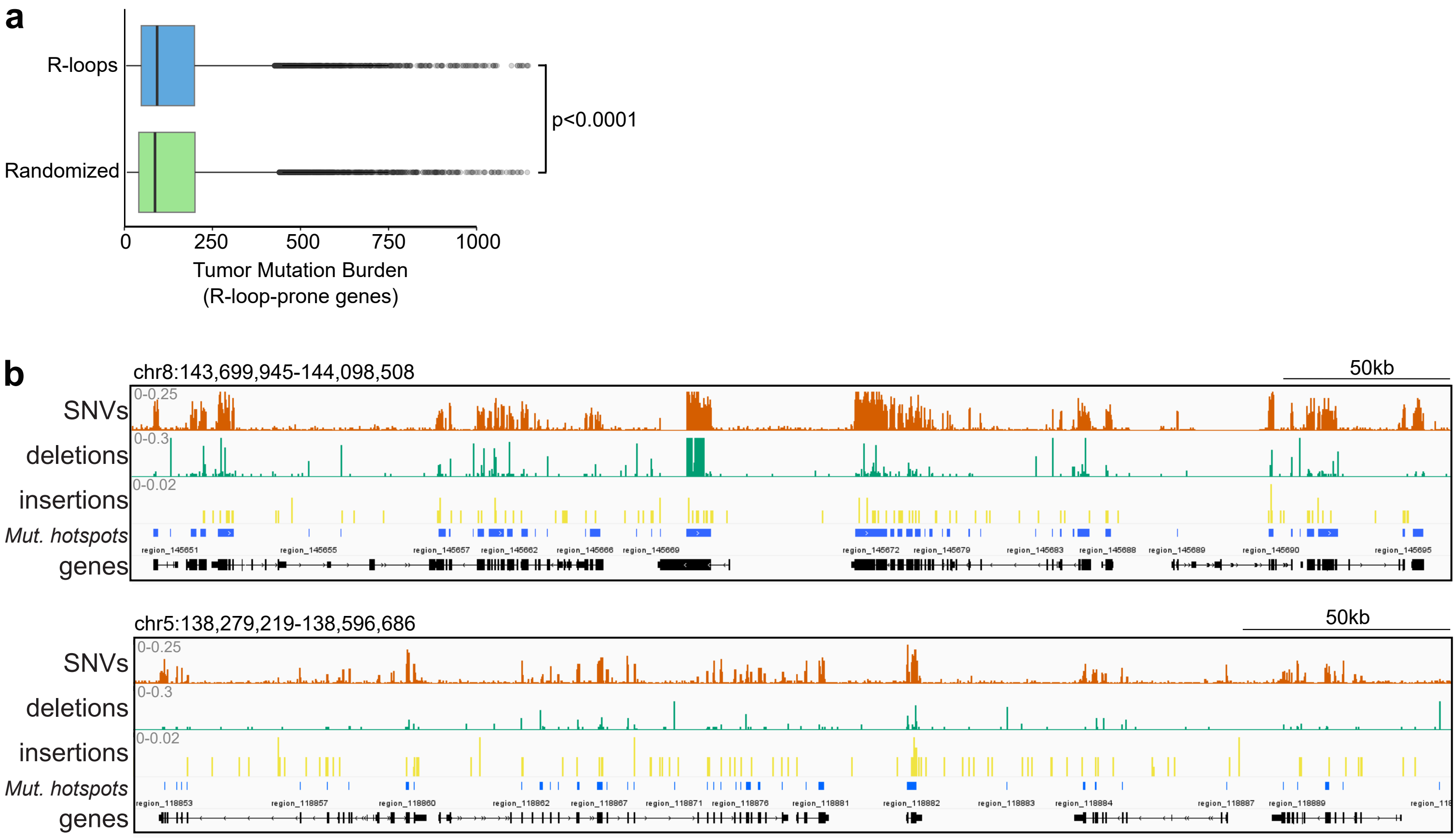

Expression:	-	+	+
R-loop:	-	-	+
Observed:	19156	39121	81722
Expected:	26759	47411	77936

P<0.0001 P<0.0001

P<0.0001

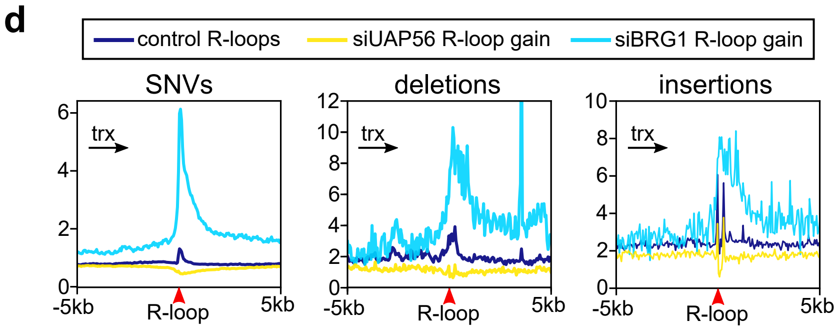

Supplementary Figure 5 – Analysis of mutational landscape at TRCs in cancer. a) Analysis of the mutation burden at R-loops. Comparison of R-loop mutation load versus randomization of the same sites along the same genes. (Wilcoxon test; two-tailed). **b)** Representative example screenshot of a genome region showing the coincidence of the identified mutation hotspots with mutations (SNVs (orange), deletions (green) and insertions (yellow)). **c)** Observed versus expected number of mutation hotspots per gene category according to the genome frequency of the different gene types analyzed. (Chi-square with Yates correction; two-tailed) **d)** Comparative metanalysis of mutational coverage at R-loop control sites, R-loop unscheduled accumulation sites in BRG1-deficient cells and UAP56-deficient cells.
P-values are indicated. SNV: Single Nucleotide Variant.

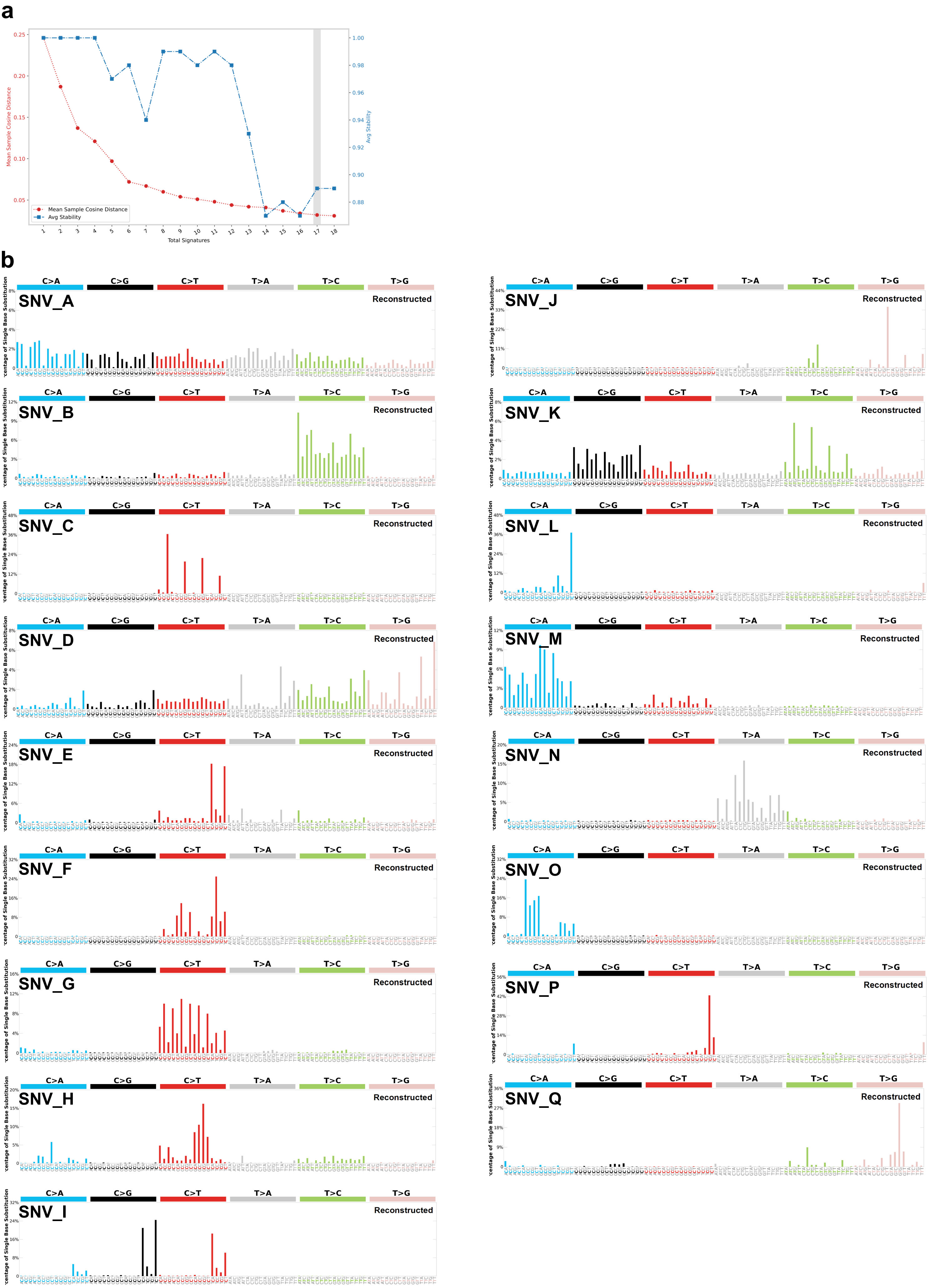

Supplementary Figure 6 – Single nucleotide variant mutational signatures extracted. a) Number of suggested solutions by SigProfiler. **b)** Plots showing the percentage of trinucleotide mutation frequencies in the mutational signatures extracted by *SigProfiler*. SNV: Single Nucleotide Variant.

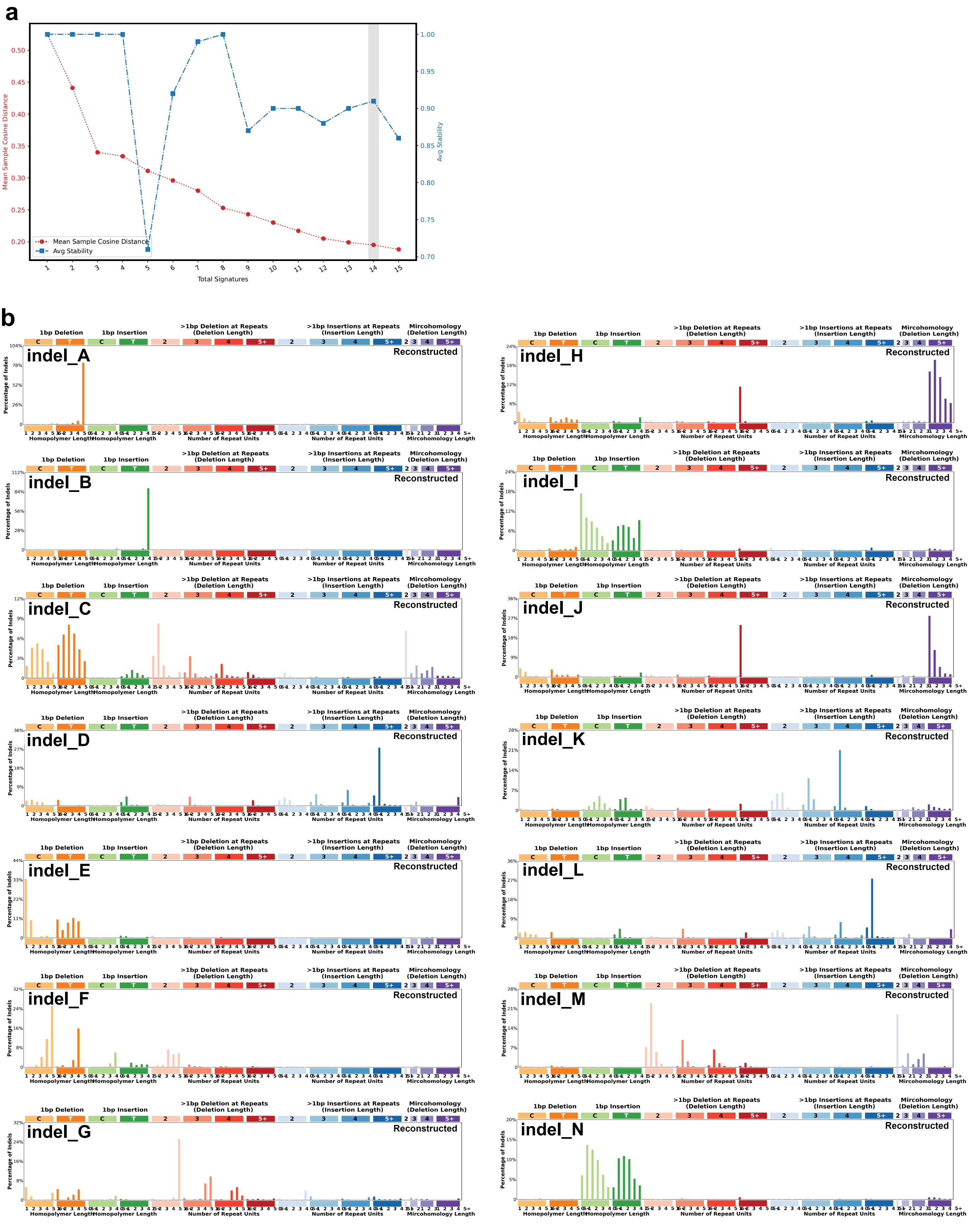

Supplementary Figure 7 – Insertion-deletion mutational signatures extracted. a) Number of suggested solutions by *SigProfiler*. **b)** Plots showing the percentage of insertion/deletion frequencies in the mutational signatures extracted by *SigProfiler*.

indel: insertion-deletion.

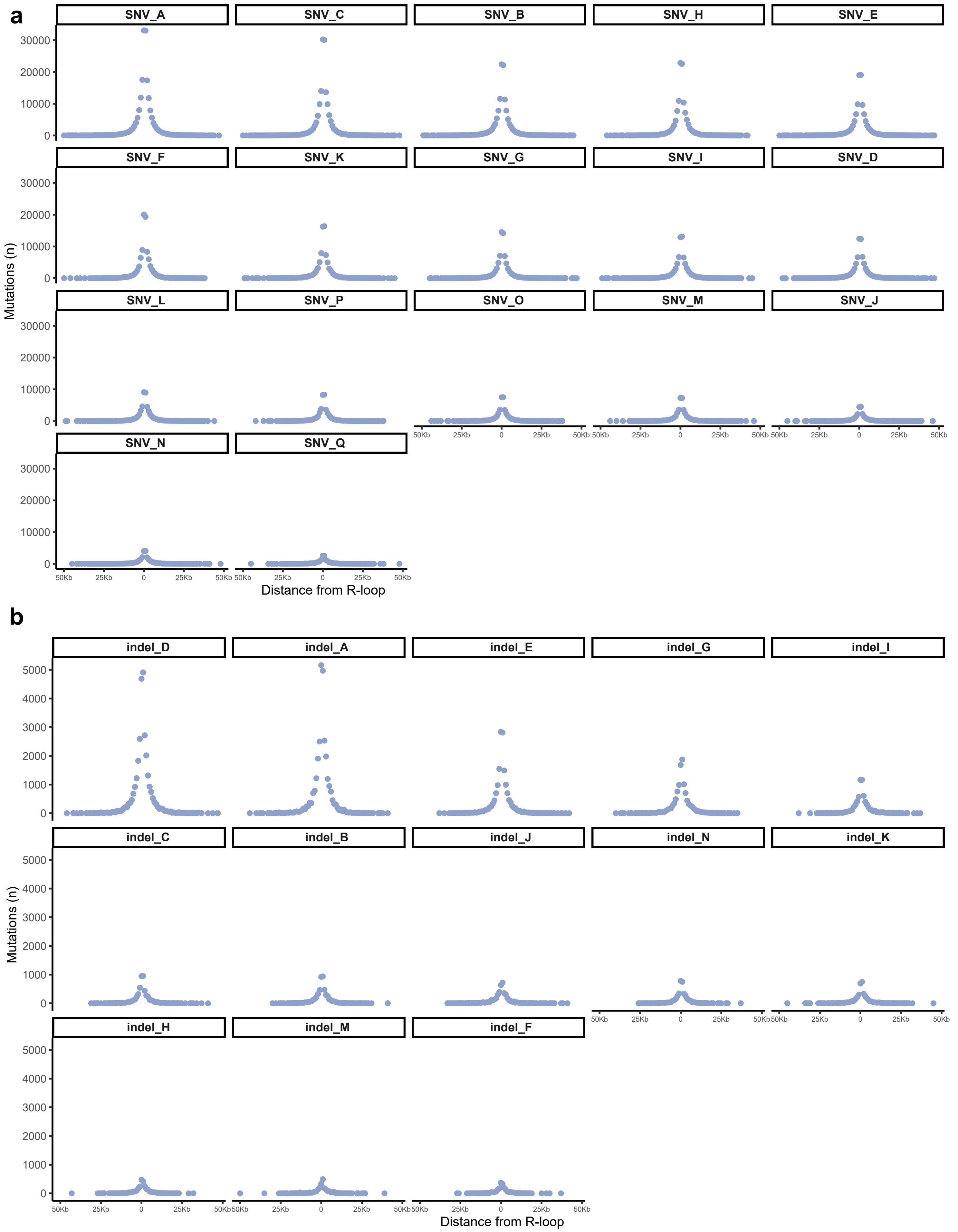

Supplementary Figure 8 – Signature profiling across R-loops. a) SNV metaplots. Number of mutations per 1kb bin around +/-50kb from R-loops for each SNV signature. **b)** Indel metaplots. Number of mutations per 1kb bin around +/-50kb from R-loops for each indel signature.

SNV: Single Nucleotide Variant; indel: insertion-deletion.

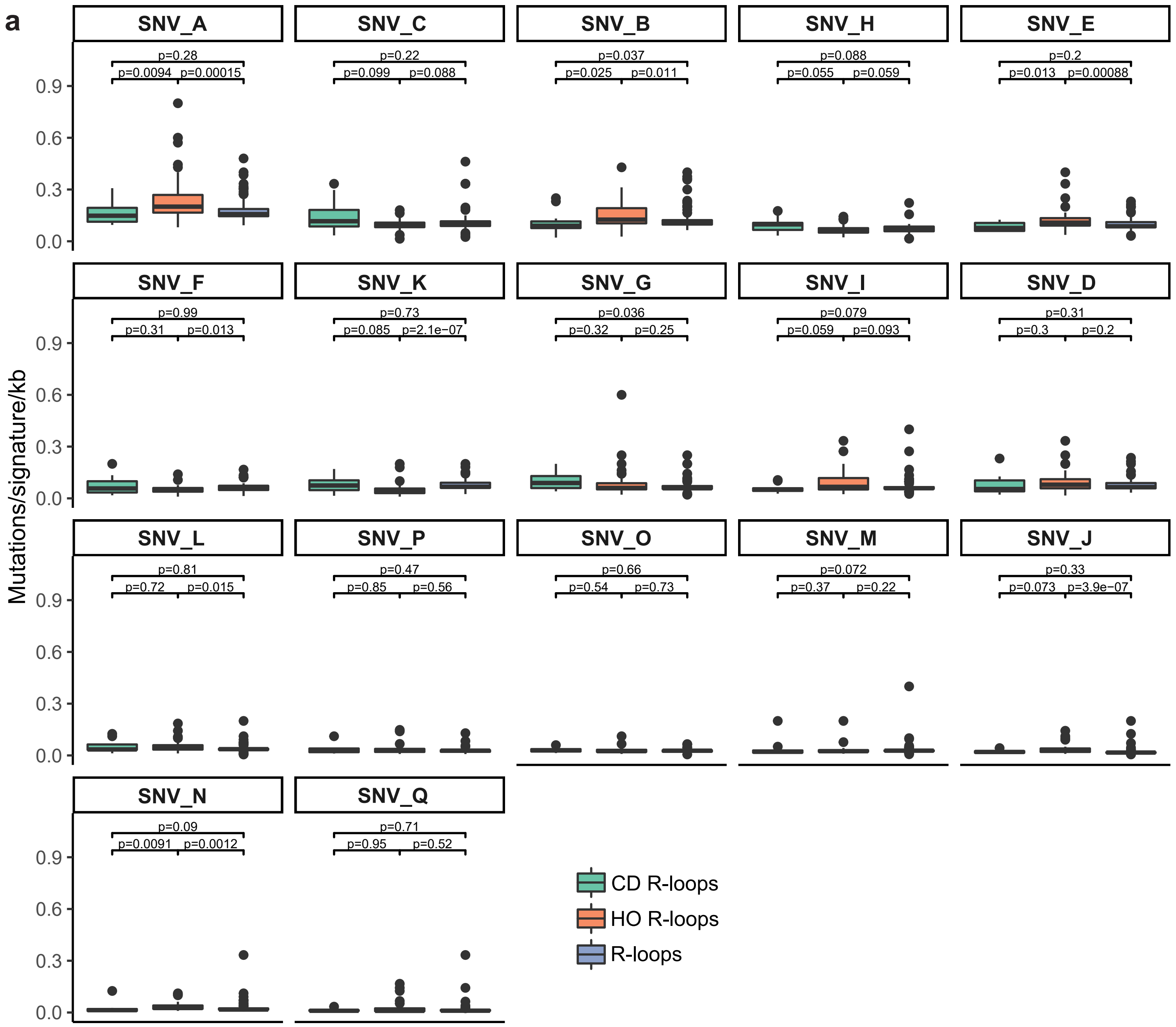

Supplementary Figure 9 – Impact of single nucleotide variant mutational signatures on head-on and co-directional TRCs. a) Frequency of mutations per kilobase bin from each SNV signature in R-loops (blue), head-on (orange) and co-directional (green) TRCs. (Wilcoxon test, two-tailed).
SNV: Single Nucleotide Variant; HO: head-on; CD: co-directional.

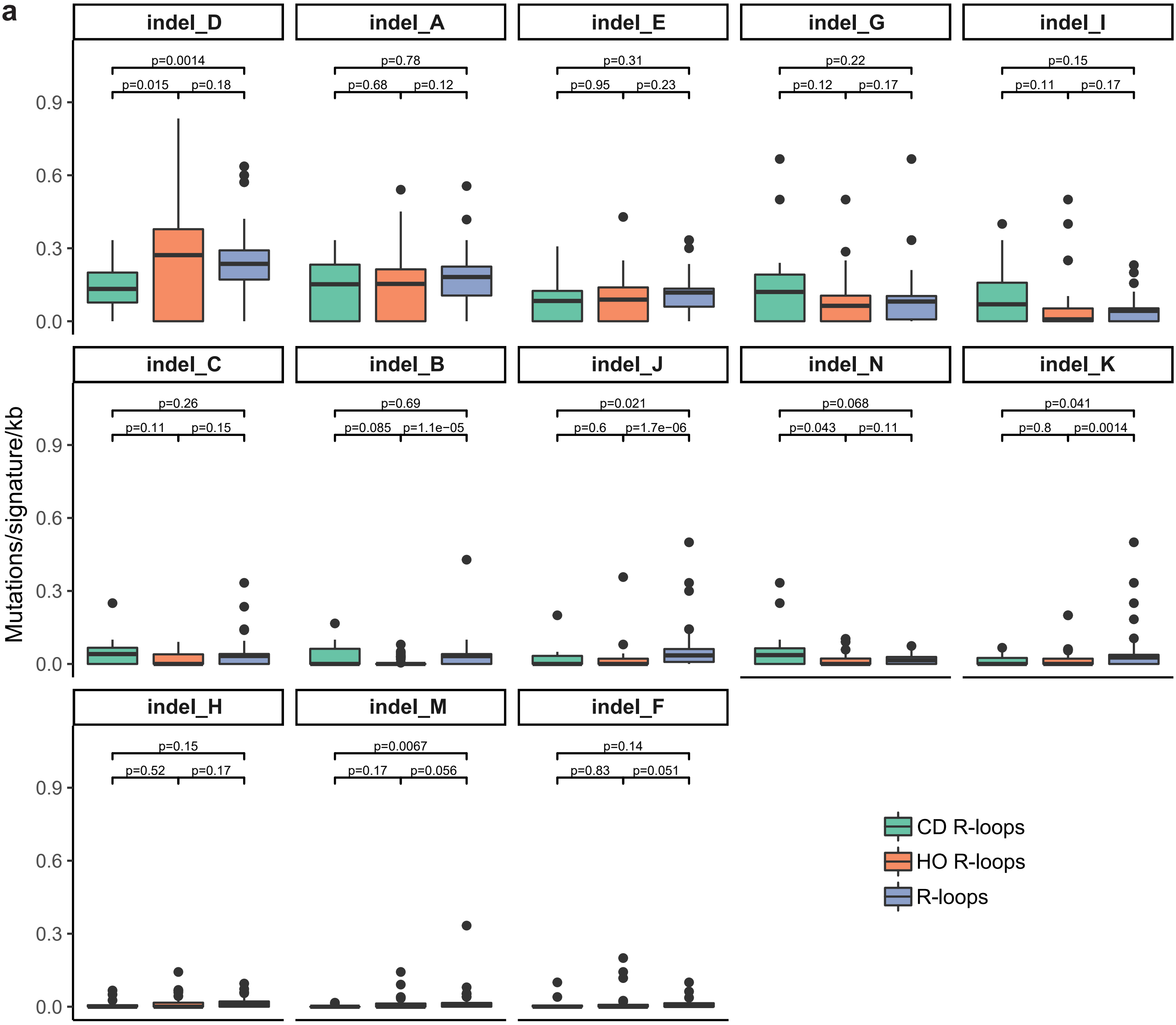

Supplementary Figure 10 – Impact of insertion-deletion mutational signatures on head-on and co-directional TRCs. a) Frequency of mutations per kilobase bin from each indel signature in R-loops (blue), head-on (orange) and co-directional (green) TRCs. (Wilcoxon test, two-tailed).
HO: head-on; CD: co-directional; indel: insertion-deletion.

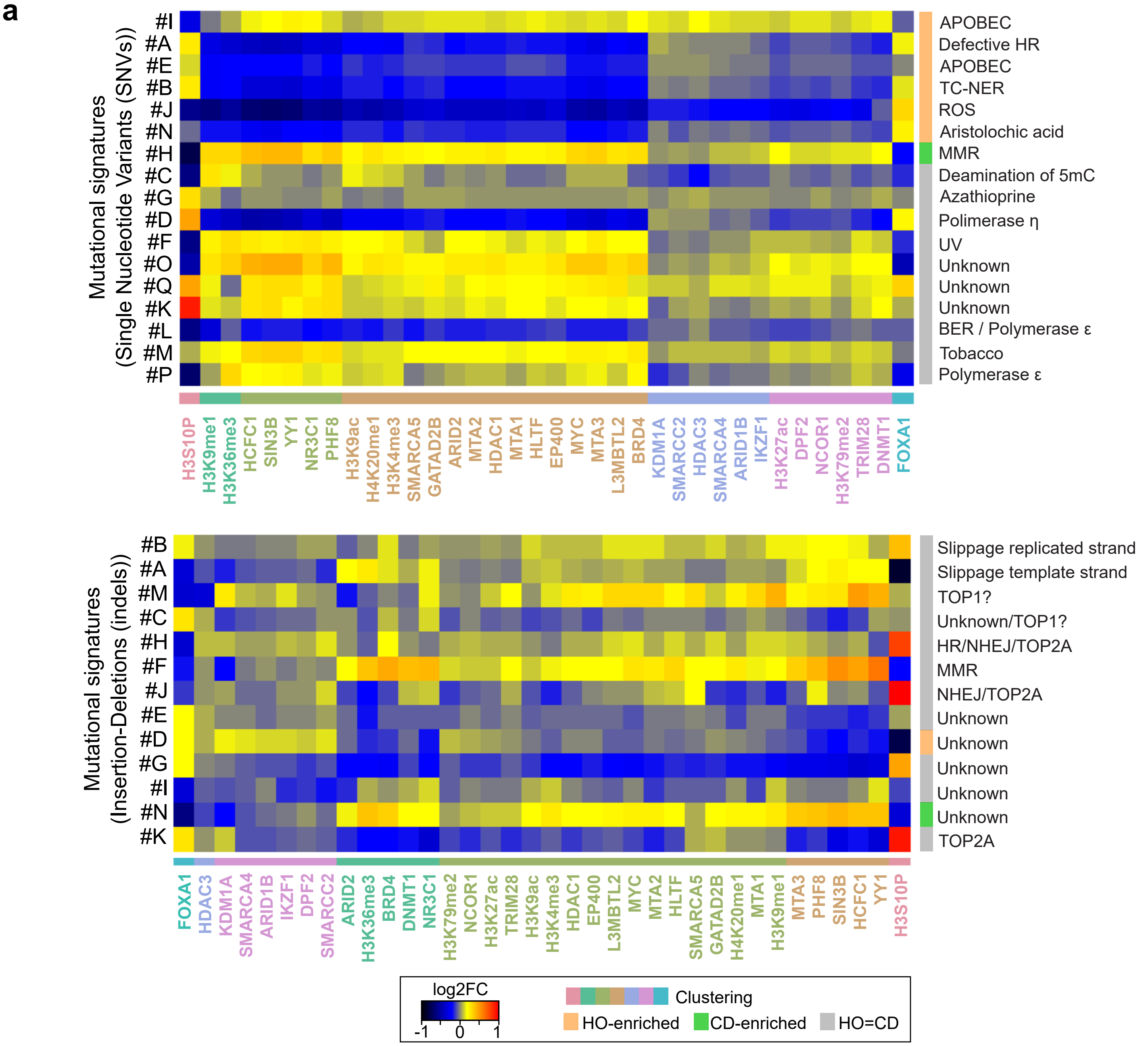

Supplementary Figure 11 – Analysis of the correlation of R-loop-associated mutational signatures and chromatin factor targeting of R-loops. a) Fold change of mutational signatures frequency in R-loop regions associated with TRC-enriched chromatin factors compared to the entire R-loop dataset (upper panel: SNVs; lower panel: indels). Data shown as heatmap. Names of mutational signatures are indicated in the left y axis and etiologies at the right y axis. Chromatin factors are specified under the x axis. Color scale values and meaning are indicated in the figure.
SNV: Single Nucleotide Variant; indel: insertion-deletion; HO: head-on; CD: co-directional.

TRC-enriched factors

Transcription by RNA polymerase II

ADNP	CC2D1A	DPF2	FOXM1	HLTF	MAFG	NCOA1	NR3C1	SIN3B	TBP	YY1	ZNF184	ZNF83
ARID1B	CDC5L	E2F1	FUS	HMBOX1	MAX	NCOA2	NRF1	SKIL	TCF12	ZBTB11	ZNF197	ZSCAN29
ARID3A	CEBPB	E2F5	GABPA	IKZF1	MAZ	NCOA6	NUFIP1	SMAD5	TCF3	ZBTB2	ZNF280A	
ARNT	CEBPG	E2F7	GABPB1	IRF1	MEIS2	NCOR1	PBX2	SMARCA4	TEAD2	ZBTB33	ZNF282	
ASH1L	COPS2	E2F8	GATAD2B	IRF9	MGA	NFATC3	PHF20	SMARCA5	TFDP1	ZBTB40	ZNF316	
ATF1	CREB1	E4F1	GMEB1	JUND	MIER1	NFE2L1	PHF8	SMARCC2	THRA	ZBTB8A	ZNF354B	
ATF2	CREB3	EGR1	GTF2A2	KAT8	MITF	NFIC	PKNOX1	SNIP1	THRAP3	ZC3H8	ZNF354C	
ATF3	CREB3L1	ELF1	GTF2E2	KDM1A	MNT	NFXL1	POLR2A	SOX6	TRIM24	ZEB2	ZNF407	
ATF4	CREM	ELF4	HCFC1	KLF13	MTA1	NKRF	POLR2B	SP1	TRIM28	ZFP91	ZNF507	
ATF6	CUX1	ETV1	HDAC1	KLF16	MTA2	NR0B1	POLR2G	SREBF2	TRIP13	ZFX	ZNF639	
ATF7	DACH1	ETV5	HDAC3	LARP7	MTA3	NR1H2	POLR2H	SRF	VEZF1	ZKSCAN8	ZNF644	
BCLAF1	DDX20	FOXA1	HDGF	LEF1	MYBL2	NR2C1	PRDM10	SUPT5H	YBX1	ZNF143	ZNF7	
BRD4	DNMT1	FOXK2	HES1	MAFF	MYC	NR2F1	RAD21	TAF9B	YBX3	ZNF148	ZNF8	

RNA polymerase complex

GTF2A2 GTF2E2 POLR2A POLR2AphosphoS2 POLR2AphosphoS5 POLR2B POLR2G POLR2H POLR3A TAF9B

Transcription initiation

BCLAF1 CREB1 GTF2A2 GTF2E2 MAZ MITF NCOA6 POLR2G SMARCA4 SMARCA5 SRF TAF9B TBP THRA

Transcription elongation

ARID1B BRD4 LARP7 SUPT5H

Transcription termination

MAZ POLR2A

RNA binding

BCLAF1	DDX20	FUS	HNRNPLL	KHSRP	NKRF	NR3C1	POLR2B	RBM22	SRF	TOE1	YBX1	ZC3H8
CDC5L	DNMT1	GTF2E2	ILF3	LARP7	NONO	NUFIP1	POLR2G	SMARCA4	SUPT5H	TRIM25	YBX3	
CSDE1	EWSR1	HDGF	KDM1A	MAZ	NR0B1	POLR2A	RBFOX2	SNIP1	THRAP3	TRIM28	YY1	

Chromatin

ADNP	CDC5L	DPF2	ETV1	HMBOX1	MAFG	MYBL2	NKRF	PML	SMARCA5	TFDP1	ZC3H8	ZNF354B
ARID1B	CEBPB	E2F1	ETV5	IKZF1	MAX	MYC	NR0B1	POLR2A	SMARCC2	THRA	ZEB2	ZNF354C
ARNT	CEBPG	E2F5	FOXA1	IRF1	MAZ	NCOA1	NR1H2	POLR2B	SOX6	TRIM24	ZKSCAN8	ZNF639
ATF1	CREB1	E2F7	FOXK2	IRF9	MEIS2	NCOA2	NR2C1	PRDM10	SP1	TRIM28	ZNF143	ZNF644
ATF2	CREB3	E2F8	FOXM1	JUND	MGA	NCOR1	NR2F1	RAD21	SREBF2	VEZF1	ZNF148	ZNF7
ATF3	CREB3L1	E4F1	GABPA	KDM1A	MITF	NFATC3	NR3C1	RAD51	SRF	YY1	ZNF184	ZNF8
ATF4	CREM	EGR1	GATAD2B	KLF13	MNT	NFE2L1	NRF1	SIN3B	TBP	ZBTB11	ZNF197	ZNF83
ATF6	CUX1	ELF1	GMEB1	KLF16	MTA1	NFIC	NUFIP1	SKIL	TCF12	ZBTB2	ZNF280A	ZSCAN29
ATF7	DACH1	ELF4	HDAC1	LEF1	MTA2	NFRKB	PBX2	SMAD5	TCF3	ZBTB33	ZNF282	
CC2D1A	DNMT1	EP400	HES1	MAFF	MTA3	NFXL1	PKNOX1	SMARCA4	TEAD2	ZBTB40	ZNF316	

Chromatin organization

ARID1B BRD4 DPF2 FOXA1 HCFC1 HDAC3 IKZF1 MTA2 NCOR1 PHF8 SMARCA5 TRIM28
ARID2 DNMT1 EP400 GATAD2B HDAC1 HLTF L3MBTL2 MYC NR3C1 SMARCA4 SMARCC2

Chromatin remodelling

ARID1B ARID2 EP400 FOXA1 GATAD2B HCFC1 HDAC1 HLTF MTA2 MYC SMARCA4 SMARCA5 SMARCC2

ATP-dependent chromatin remodellers

SMARCA4 HLTF SMARCA5 EP400

Histone modifications

H4K20me1 H3K36me3 H3K9me1 H3K27ac H3K4me2 H3K9ac H3K4me3 H3K79me2

DNA repair

ARID2 CDC5L CEBPG FANCD2 FOXM1 FUS KDM1A MTA1 NBN NFRKB NONO PML PTTG1 RAD21 RAD51 TRIM28 TRIP13 XRCC3 YY1

Double-strand break repair

ARID1A FANCD2 FOXM1 FUS KDM1A MTA1 NBN PML RAD21 RAD51 TRIP13 XRCC3 YY1

DNA damage checkpoint signalling

FANCD2 E2F1 ZNF830 CDC5L ATF2 NBN PML

Site of DNA damage

RAD51 ATF2 NBN

DNA replication

ATF1 E2F7 DACH1 RAD51 E2F8 ZNF830 E4F1 NBN NFIC

Replication fork

SMARCA5 XRCC3 DNMT1 NBN

Replication fork protection

ZNF830

DNA replication checkpoint signalling

ZNF830

Replication fork processing

RAD51

Supplementary Table 1 – TRC-enriched factors.
Table containing genes detected as enriched at TRCs in our experimental approach. Genes are classified according to GO term. Bold text is used to highlight GO terms. TRC: transcription-replication conflict.

TRC-depleted factors
**Transcription by RNA polymerase II**
CBX8 CTCFL ETS1 THAP1 ZBTB7A<br>**Transcription termination**<br>PRMT5
**RNA binding**
CBX8
**Chromatin**
CBX8 CTCFL ETS1 THAP1 ZBTB7A PRMT5<br>**Chromatin organization**<br>ZBTB7A<br>**Chromatin remodelling**<br>ZBTB7A<br>**Histone modifications**<br>H3K27me3
**DNA Repair**
CBX8 ZBTB7A<br>**Double-strand break repair**<br>ZBTB7A<br>**Site of DNA damage**<br>ZBTB7A
**DNA replication**
MCM2 MCM3 MCM5 MCM7<br>**Replication fork**<br>MCM3<br>**MCM complex**<br>MCM2 MCM3 MCM5 MCM7<br>**DNA helicases**<br>MCM3

Supplementary Table 2 – TRC-depleted factors.
Table containing genes detected as depleted at TRCs in our experimental approach. Genes are classified according to GO term. Bold text is used to highlight GO terms. TRC: transcription-replication conflict.

HO-enriched factors

Transcription by RNA polymerase II

YY1	ZNF7	ZFX	PHF8	GABPA	NR2F1	CREB3L1	ZBTB2	MAFG	NFXL1	RAD21	POLR2H
BRD4	MYC	MAX	ZNF148	IKZF1	MEIS2	GTF2A2	SMARCC2	POLR2G	DACH1	FOXK2	NR2C1
TBP	ETV5	ARID3A	TAF9B	ZNF407	MTA1	BCLAF1	ZNF316	PBX2	ZBTB40	ZNF354C	ATF7
ZNF143	MAFF	E2F5	ZNF83	SRF	DPF2	ATF1	ATF6	LEF1	ZBTB8A	NR0B1	PKNOX1
CUX1	IRF9	ATF4	NFE2L1	MAZ	HDAC1	HLTF	MITF	PRDM10	CDC5L	GATAD2B	DDX20
JUND	ELF1	SOX6	SMARCA4	ZFP91	GTF2E2	MIER1	YBX3	NFATC3	ZBTB33	POLR2A	KDM1A
FUS	CEBPB	ZNF644	TRIM24	TFDP1	LARP7	ZC3H8	ZEB2	NCOA2	NUFIP1	E2F8	MNT
ZNF354B	HCFC1	ZNF197	HMBOX1	SKIL	CC2D1A	ELF4	YBX1	GABPB1	THRA	HDAC3	POLR2B
KLF13	PHF20	TCF3	MTA2	ZSCAN29	NR1H2	E2F7	SMARCA5	ZNF8	ASH1L	GMEB1	
TEAD2	CREB3	ZNF184	SMAD5	ADNP	SREBF2	NKRF	HES1	NR3C1	NRF1	MYBL2	
VEZF1	ZNF639	CEBPG	ETV1	CREB1	SNIP1	TRIM28	TRIP13	KLF16	HDGF	NCOA6	
CREM	TCF12	ZKSCAN8	NCOR1	E2F1	ARID1B	SIN3B	ZNF280A	NCOA1	DNMT1	THRAP3	
SP1	EGR1	ZBTB11	ZNF282	MTA3	MGA	ARNT	ATF3	KAT8	FOXM1	E4F1	

RNA polymerase complex
GTF2A2 GTF2E2 POLR2A POLR2B POLR2G POLR2H POLR3A TAF9B

Transcription initiation
BCLAF1 CREB1 GTF2A2 GTF2E2 MAZ MITF NCOA6 POLR2G SMARCA4 SMARCA5 SRF TAF9B TBP THRA

Transcription elongation
ARID1B BRD4 LARP7

Transcription termination
MAZ POLR2A

RNA binding

BCLAF1	DDX20	FUS	ILF3	LARP7	NONO	NUFIP1	POLR2G	SMARCA4	THRAP3	TRIM28	YY1
CDC5L	DNMT1	GTF2E2	KDM1A	MAZ	NR0B1	POLR2A	RBFOX2	SNIP1	TOE1	YBX1	ZC3H8
CSDE1	EWSR1	HDGF	KHSRP	NKRF	NR3C1	POLR2B	RBM22	SRF	TRIM25	YBX3	

Chromatin

YY1	SP1	TCF12	CEBPG	SMAD5	ZSCAN29	NFRKB	E2F7	ZEB2	NCOA2	NUFIP1	NR0B1	KDM1A
TBP	ZNF7	EGR1	ZKSCAN8	ETV1	ADNP	CC2D1A	NKRF	SMARCA5	ZNF8	THRA	GATAD2B	MNT
ZNF143	MYC	MAX	ZBTB11	NCOR1	CREB1	NR1H2	TRIM28	HES1	NR3C1	NRF1	POLR2A	POLR2B
CUX1	ETV5	E2F5	ZNF148	ZNF282	E2F1	SREBF2	SIN3B	ZNF280A	KLF16	DNMT1	E2F8	
JUND	MAFF	ATF4	ZNF83	GABPA	MTA3	ARID1B	ARNT	ATF3	NCOA1	EP400	GMEB1	
ZNF354B	IRF9	SOX6	NFE2L1	IKZF1	NR2F1	MGA	ZBTB2	MAFG	NFXL1	FOXM1	MYBL2	
KLF13	ELF1	ZNF644	SMARCA4	SRF	MEIS2	CREB3L1	SMARCC2	PBX2	DACH1	RAD21	E4F1	
TEAD2	CEBPB	ZNF197	TRIM24	MAZ	MTA1	ATF1	ZNF316	LEF1	ZBTB40	FOXK2	NR2C1	
VEZF1	CREB3	TCF3	HMBOX1	TFDP1	DPF2	ZC3H8	ATF6	PRDM10	CDC5L	ZNF354C	ATF7	
CREM	ZNF639	ZNF184	MTA2	SKIL	HDAC1	ELF4	MITF	NFATC3	ZBTB33	RAD51	PKNOX1	

Chromatin organization

ARID1B	BRD4	DPF2	GATAD2B	HDAC1	HLTF	L3MBTL2	MYC	NR3C1	SMARCA4	SMARCC2
ARID2	DNMT1	EP400	HCFC1	HDAC3	IKZF1	MTA2	NCOR1	PHF8	SMARCA5	TRIM28

Chromatin remodelling
ARID1B ARID2 EP400 GATAD2B HCFC1 HDAC1 HLTF MTA2 MYC SMARCA4 SMARCA5 SMARCC2

ATP-dependent chromatin remodellers
SMARCA4 HLTF SMARCA5 EP400

Histone modifications
H4K20me1 H3K9me1 H3K27ac H3K4me2 H3K9ac H3K4me3 H3K79me2

DNA repair

ARID2 CDC5L CEBPG FANCD2 FOXM1 FUS KDM1A MTA1 NFRKB NONO PTTG1 RAD21 RAD51 TRIM28 TRIP13 XRCC3 YY1

Double-strand break repair
ARID2 FANCD2 FOXM1 FUS KDM1A MTA1 RAD21 RAD51 TRIP13 XRCC3 YY1

DNA damage checkpoint signalling
FANCD2 E2F1 ZNF830 CDC5L

Site of DNA damage
RAD51

DNA replication

ATF1 E2F7 DACH1 RAD51 E2F8 ZNF830 E4F1

Replication fork
SMARCA5 XRCC3 DNMT1

Replication fork protection
ZNF830

DNA replication checkpoint signalling
ZNF830

Replication fork processing
RAD51

Supplementary Table 3 – Genes enriched at head-on TRCs.

Table containing genes detected as increased at head-on TRCs. Genes are classified according to GO term. Bold text is used to highlight GO terms. HO: head-on.

CD-enriched factors
**Transcription by RNA polymerase II**
FOXA1 COPS2 *THAP1* *ETS1* *CTCFL* *ZBTB7A* *CBX8* SUPT5H<br>**Transcription elongation**<br>SUPT5H<br>**Transcription termination**<br>*PRMT5*
**RNA binding**
CBX8 HNRNPLL SUPT5H
**Chromatin**
FOXA1 PML *THAP1* *ETS1* *CTCFL* *ZBTB7A* *PRMT5* *CBX8*<br>**Chromatin organization**<br>*ZBTB7A* FOXA1<br>**Chromatin remodelling**<br>*ZBTB7A* FOXA1<br>**Histone modifications**<br>*H3K27me3*
**DNA Repair**
CBX8 *ZBTB7A* PML<br>**Double-strand break repair**<br>*ZBTB7A* PML<br>**Site of DNA damage**<br>*ZBTB7A*<br>**DNA damage checkpoint signalling**<br>PML
**DNA replication**
MCM2 *MCM3* *MCM5* *MCM7*<br>**MCM complex**<br>MCM2 *MCM3* *MCM5* *MCM7*<br>**DNA helicase activity**<br>*MCM3*<br>**Replication fork**<br>*MCM3*

Supplementary Table 4 – Genes enriched at co-directional TRCs.

Table containing genes detected as increased at co-directional TRCs. Genes are classified according to GO term. Bold text is used to highlight GO terms. TRC-depleted proteins are highlighted in italics. CD: co-directional.

Signature ID	Cosine Similarity	Correlation	COSMIC mutational signature matches
**SNV_A**	0.924	0.847	SBS3 (68.7%); Defective homologous recombination-based DNA damage repair<br>SBS8 (28.8%); Unknown<br>SBS1 (2.6%); Spontaneous or enzymatic deamination of 5-methylcytosine
**SNV_B**	0.984	0.98	SBS12 (77.6%); Transcription-coupled nucleotide excision repair<br>SBS16 (18.22%); Transcription-coupled nucleotide excision repair<br>SBS5 (4.16%); Unknown<br>SBS1 (0.02%); Spontaneous or enzymatic deamination of 5-methylcytosine
**SNV_C**	0.991	0.991	SBS1; Spontaneous or enzymatic deamination of 5-methylcytosine
**SNV_D**	0.893	0.821	SBS9 (59.18%); Polimerase eta somatic hypermutation activity<br>SBS41 (34.18%); Unknown<br>SBS5 (5.46%); Unknown<br>SBS1 (1.18%); Spontaneous or enzymatic deamination of 5-methylcytosine
**SNV_E**	0.987	0.985	SBS58 (40.46%); Potential sequencing artefact<br>SBS2 (33.22%); Activity of the AID/APOBEC family of cytidine deaminases<br>SBS34 (12.46%); Unknown<br>SBS5 (10.38%); Unknown<br>SBS1 (3.48%); Spontaneous or enzymatic deamination of 5-methylcytosine
**SNV_F**	0.984	0.983	SBS7b (65.96%); Ultraviolet light exposure<br>SBS7a (33.18%); Ultraviolet light exposure<br>SBS1 (0.86%); Spontaneous or enzymatic deamination of 5-methylcytosine
**SNV_G**	0.949	0.943	SBS23 (50.56%); Unknown<br>SBS32 (47.64%); Azathioprine treatment<br>SBS5 (1.8%); Unknown
**SNV_H**	0.928	0.915	SBS44 (50.62%); Defective DNA mismatch repair<br>SBS15 (49.38%); Defective DNA mismatch repair
**SNV_I**	0.994	0.994	SBS13 (66.0%); Activity of the AID/APOBEC family of cytidine deaminases<br>SBS2 (33.66%); Activity of the AID/APOBEC family of cytidine deaminases<br>SBS1 (0.34%); Spontaneous or enzymatic deamination of 5-methylcytosine
**SNV_J**	0.995	0.995	SBS17b (62.76%); 5-FU (fluorouracil) chemotherapy<br>SBS17a (28.24%); DNA damage by ROS<br>SBS5 (9.0%); Unknown
**SNV_K**	0.864	0.735	SBS39 (62.76%); Unknown<br>SBS54 (24.56%); Potential sequencing artefact<br>SBS5 (5.76%); Unknown<br>SBS1 (2.46%); Spontaneous or enzymatic deamination of 5-methylcytosine
**SNV_L**	0.997	0.997	SBS36 (46.46%); Defective base excision repair due to MUTYH mutations<br>SBS10a (40.96%); Polymerase epsilon exonuclease domain mutations<br>SBS28 (10.46%); Unknown<br>SBS1 (2.12%); Spontaneous or enzymatic deamination of 5-methylcytosine
**SNV_M**	0.907	0.897	SBS29; Tobacco chewing
**SNV_N**	0.984	0.981	SBS22 (94.42%); Aristolochic acid exposure<br>SBS5 (5.58%); Unknown
**SNV_O**	0.987	0.988	SBS45 (81.76%); Possible artefact due to 8-oxo-guanine<br>SBS49 (18.24%); Possible sequencing artefact
**SNV_P**	0.972	0.972	SBS10b (97.1%); Polymerase epsilon exonuclease domain mutations<br>SBS1 (2.9%); Spontaneous or enzymatic deamination of 5-methylcytosine
**SNV_Q**	0.987	0.986	SBS43 (84.48%); Possible sequencing artefact<br>SBS60 (13.5%); Possible sequencing artefact<br>SBS5 (2.02%); Unknown

Supplementary Table 5 – Single nucleotide variant signatures identified at R-loop sites and similarity to COSMIC reference signatures.

List of single nucleotide variant signatures extracted and similarity to COSMIC signatures. SNV: Single Nucleotide Variant; SBS: Single Base Substitution.

Signature ID	Cosine Similarity	Correlation	COSMIC mutational signature matches
**indel_A**	0.997	0.997	ID2; Slippage during DNA replication of the template DNA strand
**indel_B**	0.999	0.999	ID1; Slippage during DNA replication of the replicated DNA strand
**indel_C**	0.892	0.854	ID5 (40.82%); Unknown<br>ID4 (31.54%); TOP1?<br>ID15 (27.64%); Unknown
**indel_D**	0.236	0.138	ID10; Unknown
**indel_E**	0.902	0.894	ID9; Unknown
**indel_F**	0.928	0.925	ID7; Defective DNA mismatch reapir
**indel_G**	0.843	0.86	ID12; Unknown
**indel_H**	0.986	0.986	ID6 (57.08%); Defective homologous recombination-based DNA damage repair<br>ID8 (42.92%); Repair of DSBs by NHEJ or mutations in TOP2A
**indel_I**	0.729	0.689	ID16 (70.94%); Unknown<br>ID14 (29.06%); Unknown
**indel_J**	0.311	0.279	ID8; Repair of DSBs by NHEJ or mutations in TOP2A
**indel_K**	0.151	0.087	ID17; Mutations in topoisomerase TOP2A
**indel_L**	0.498	0.408	ID10; Unknown
**indel_M**	0.39	0.336	ID4; TOP1?
**indel_N**	0.259	0.21	ID16; Unknown

Supplementary Table 6 – Insertion-deletion signatures identified at R-loop sites and similarity to COSMIC reference signature.

List of insertion-deletion signatures extracted and similarity to COSMIC signatures. indel: insertion-deletion; ID: insertion-deletion.

siRNAs		
Name	Source	Identifier
ON-TARGETplus Non-targeting Control Pool	Dharmacon	D-001810-10
ON-TARGETplus Human SMARCA4	Dharmacon	L-010431-00
ON-TARGETplus Human SMARCA5	Dharmacon	L-011478-00
ON-TARGETplus Human INO80	Dharmacon	L-004176-01
ON-TARGETplus Human MTA2	Dharmacon	L-008482-00

Supplementary Table 7 – siRNAs used for protein knock-down.
Table containing siRNAs used for protein depletion. Names, sources and identifiers are indicated.

DNA primers	
Name	Sequence
FOXP4-Fwd	5’-TTGGTGCACGTGGTTTTCTC-3’
FOXP4-Rv	5’-CCTAAAGCAGGTGCAGCAACT-3’
RPL13A-Fwd	5’-GCTTCCAGCACAGGACAGGTAT-3
RPL13A-Rv	5’-CACCCACTACCCGAGTTCAAG-3’
TAF9B-Fwd	5'-AAACCCAACTGCTCTGACATAAGA-3'
TAF9B-Rv	5'-TGCTCTACATGCCTAATGTTTTGTG-3'
5'rDNA-Fwd	5’-GGTATATCTTTCGCTCCGAGTC-3’
5'rDNA-Rv	5’-GGACAGCGTGTCAGCAATAA-3’
28SrDNA-Fwd	5'-GAATCCGCTAAGGAGTGTGTAACA-3'
28SrDNA-Rv	5'-CTCCAGCGCCATCCATTT-3'

Supplementary Table 8 – DNA primers used for qPCRs in DRIP analyses.
Table containing primer sequences used in DRIP-qPCR experiments.

Uncropped scans of blots shown is Supplementary Figure 3.

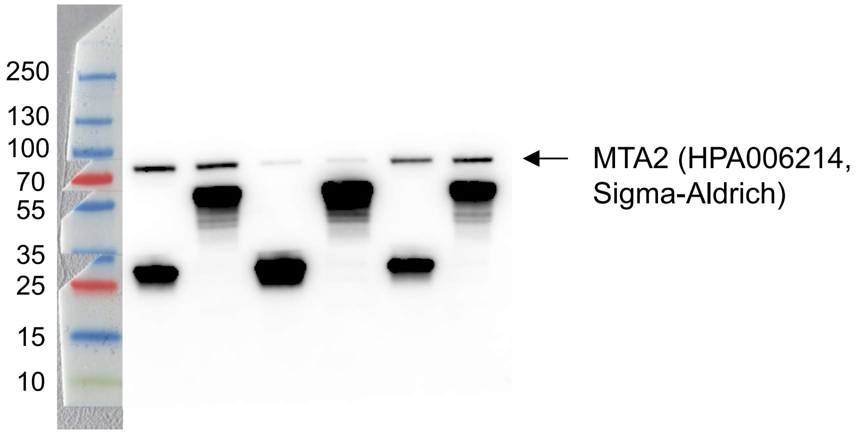

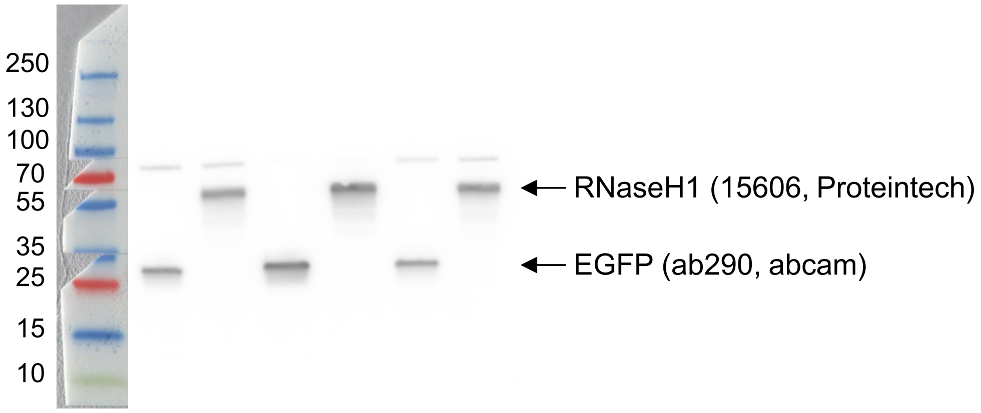

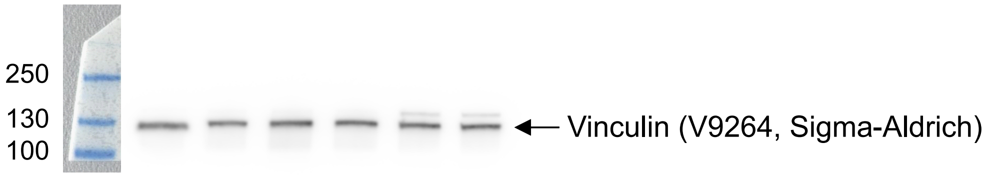

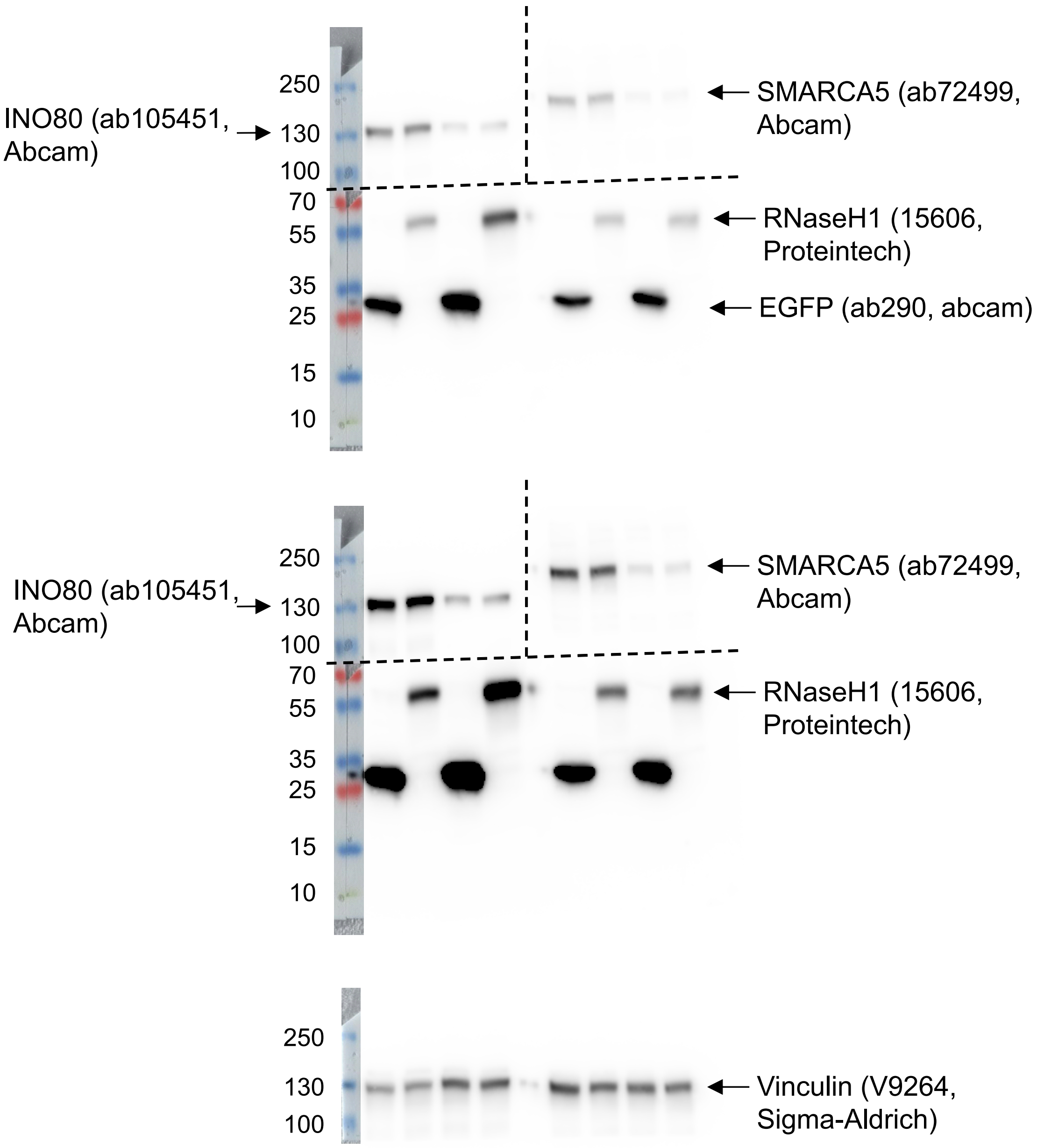
INO80 (ab105451, Abcam)
250
130
100
70
55
35
25
15
10
SMARCA5 (ab72499, Abcam)
RNaseH1 (15606, Proteintech)
EGFP (ab290, abcam)
INO80 (ab105451, Abcam)
250
130
100
70
55
35
25
15
10
SMARCA5 (ab72499, Abcam)
RNaseH1 (15606, Proteintech)
250
130
100
Vinculin (V9264, Sigma-Aldrich)

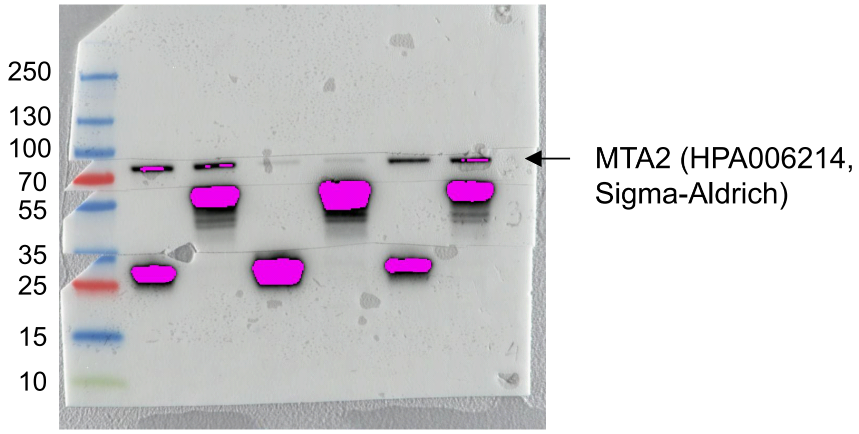
250
130
100
70
55
35
25
15
10
MTA2 (HPA006214, Sigma-Aldrich)

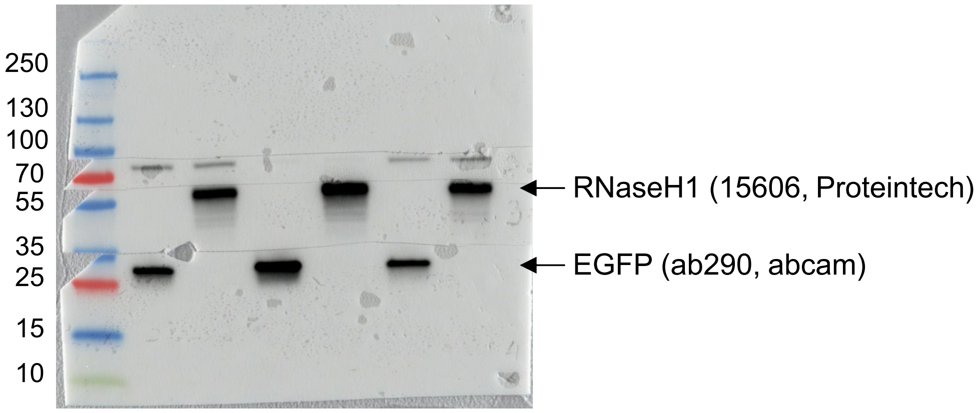
250
130
100
70
55
35
25
15
10
RNaseH1 (15606, Proteintech)
EGFP (ab290, abcam)

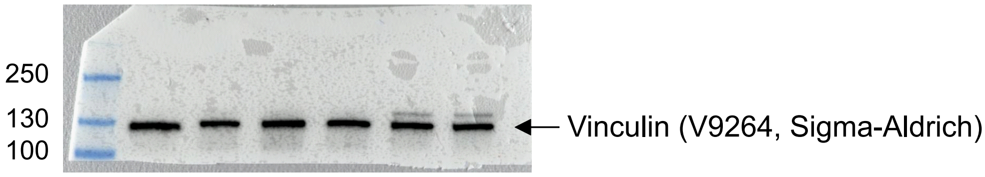
250
130
100
Vinculin (V9264, Sigma-Aldrich)

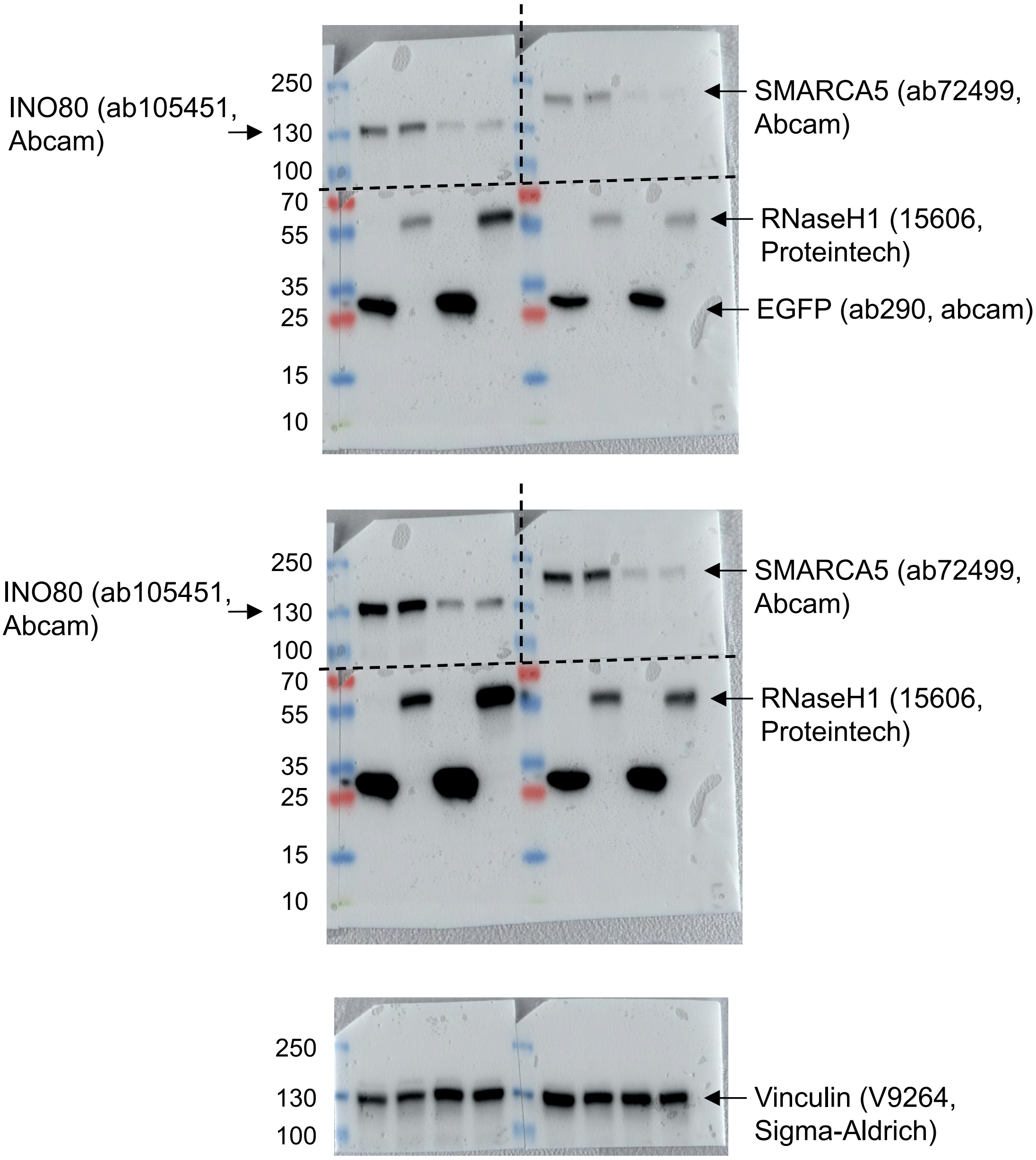

INO80 (ab105451, Abcam)
250
130
100
70
55
35
25
15
10
SMARCA5 (ab72499, Abcam)
RNaseH1 (15606, Proteintech)
EGFP (ab290, abcam)
INO80 (ab105451, Abcam)
250
130
100
70
55
35
25
15
10
SMARCA5 (ab72499, Abcam)
RNaseH1 (15606, Proteintech)
250
130
100
Vinculin (V9264, Sigma-Aldrich)
