# Supplementary material for: The chromatin network helps prevent cancer-associated mutagenesis at transcription-replication conflicts
Source: Nat Commun. 2023 Oct 28;14:6890. doi: 10.1038/s41467-023-42653-0 (PMC10613258; doi:10.1038/s41467-023-42653-0)
Supplement: Supplementary file 3 — Reporting Summary [file 41467_2023_42653_MOESM3_ESM.pdf]

nature portfolio

Corresponding author(s): Andrés Aguilera; Aleix Bayona-Feliu

Last updated by author(s): Sep 29, 2023

Reporting Summary

Nature Portfolio wishes to improve the reproducibility of the work that we publish. This form provides structure for consistency and transparency in reporting. For further information on Nature Portfolio policies, see our Editorial Policies and the Editorial Policy Checklist.

Statistics

For all statistical analyses, confirm that the following items are present in the figure legend, table legend, main text, or Methods section.

n/a	Confirmed	
☐	☒	The exact sample size (*n*) for each experimental group/condition, given as a discrete number and unit of measurement
☐	☒	A statement on whether measurements were taken from distinct samples or whether the same sample was measured repeatedly
☐	☒	The statistical test(s) used AND whether they are one- or two-sided<br>*Only common tests should be described solely by name; describe more complex techniques in the Methods section.*
☒	☐	A description of all covariates tested
☒	☐	A description of any assumptions or corrections, such as tests of normality and adjustment for multiple comparisons
☐	☒	A full description of the statistical parameters including central tendency (e.g. means) or other basic estimates (e.g. regression coefficient) AND variation (e.g. standard deviation) or associated estimates of uncertainty (e.g. confidence intervals)
☐	☒	For null hypothesis testing, the test statistic (e.g. *F*, *t*, *r*) with confidence intervals, effect sizes, degrees of freedom and *P* value noted<br>*Give P values as exact values whenever suitable.*
☒	☐	For Bayesian analysis, information on the choice of priors and Markov chain Monte Carlo settings
☒	☐	For hierarchical and complex designs, identification of the appropriate level for tests and full reporting of outcomes
☒	☐	Estimates of effect sizes (e.g. Cohen's *d*, Pearson's *r*), indicating how they were calculated

Our web collection on statistics for biologists contains articles on many of the points above.

Software and code

Policy information about availability of computer code

Data collection	Immunofluorescence images were acquired with a Leica DM6000 microscope equipped with a DFC390 camera and LAS AX v2.0 image acquisition software (Leica). Immunofluorescence data quantification was performed using FIJI (Fiji Is Just ImageJ) image processing package v2.0.0-rc-69/1.52p.<br>Western blot images were acquired using AMERSHAM ImageQuant 800 (GE Healthcare).<br>Quantitative PCR results were obtained from a 7500 FAST Real-Time PCR System equipped with 7500 Software v2.3.
Data analysis	Downstream processing of ChIP-seq data was performed using the European Galaxy Platform v18.05-20.05 (https://usegalaxy.eu), UNIX command line (GNU bash, version 4.2.46(2)) and R studio (2022.07.0 Build 548) with R 4.2.1 version. The following software and algorithms were used:<br>SAMtools (up to Galaxy Version 2.0.3 / UNIX 1.9)<br>deeptools (up to Galaxy Version 3.3.2.0.0 / UNIX 3.5.1)<br>BEDtools (up to Galaxy Version 2.29.2 / UNIX 2.29.2)<br>MACS2 (up to Galaxy Version 2.1.1.20160309.6)<br>ChIPseeker (up to Galaxy Version 1.18.0+galaxy1)<br>BEDOPS (UNIX 2.4.35)<br>HOMER (UNIX v4.11)<br>UCSC tools (bedGraphToBigWig) (UNIX v4)<br>SigProfilerMatrixGenerator (UNIX 1.1.28)<br>SigProfilerExtractor (UNIX 1.0.14)<br>Visualization of genome-wide tracks was performed on Integrative Genome Viewer v2.15.2.<br>Codirectional and head-on average contact matrix were built using the R script deposited in the Github Repository (https://github.com/

eusololi/combined_rloops_hic_map.git; DOI: 10.5281/zenodo.8359279).
Immunoflurescence image processing was performed using FIJI (Fiji Is Just ImageJ) image processing package v2.0.0-rc-69/1.52p. FIJI macros for S9.6 mean nuclear intensity and gammaH2AX foci quantification were deposited in the Zenodo repository (https://zenodo.org/record/8390817; DOI: 10.5281/zenodo.8390817). For immunofluorescence analysis along the cell cycle, the DNA cell cycle FIJI Plug-in 2013/07/07: Updated to December 2009 release was used.
Calculations, histograms, box and scatter plots were performed using Microsoft Excel 2016 (16.0.5404.1002) MSO (16.0.5404.1000) 64-bit and GraphPad Prism v9. HiC results were plotted using Gitools v2.3.x.

For manuscripts utilizing custom algorithms or software that are central to the research but not yet described in published literature, software must be made available to editors and reviewers. We strongly encourage code deposition in a community repository (e.g. GitHub). See the Nature Portfolio guidelines for submitting code & software for further information.

Data

Policy information about availability of data

All manuscripts must include a data availability statement. This statement should provide the following information, where applicable:

- Accession codes, unique identifiers, or web links for publicly available datasets
- A description of any restrictions on data availability
- For clinical datasets or third party data, please ensure that the statement adheres to our policy

No datasets were generated during the current study. The data collected and used in this study are available as open data in the following repositories:
a) ENCODE ChIP-seq collection
The entire collection of ChIP-seq datasets available in The ENCODE Project repository (https://www.encodeproject.org/) (Feingold, E. A. et al. Science 2004) were used for this study. A detailed full list with the accession numbers of the datasets used is available as Supplementary Data.
b) DRIPc-seq
GEO: GSE154631/GSE127979
(https://www.ncbi.nlm.nih.gov/geo/query/acc.cgi?acc=GSE154631 ; https://www.ncbi.nlm.nih.gov/geo/query/acc.cgi?acc=GSE127979)
c) FANCD2 ChIP-seq
NCBI SRA: PRJNA473287
(https://www.ncbi.nlm.nih.gov/bioproject/?term=PRJNA473287)
d) H3S10pho ChIP-seq
GEO: GSE144288
(https://www.ncbi.nlm.nih.gov/geo/query/acc.cgi?acc=GSE144288)
e) OK-seq
EMBL-EBI ENA: PRJEB25180
(https://www.ebi.ac.uk/ena/browser/view/PRJEB25180)
The cancer mutation data used in this study was entirely obtained from the COMSIC database (https://cancer.sanger.ac.uk/cosmic/download).
f) RNA-seq
GEO: GSE154631/GSE127979
(https://www.ncbi.nlm.nih.gov/geo/query/acc.cgi?acc=GSE154631 ; https://www.ncbi.nlm.nih.gov/geo/query/acc.cgi?acc=GSE127979)
g) Cancer mutation data
Cancer mutation data were retrieved from the COSMIC database (https://cancer.sanger.ac.uk/cosmic/download) (Tate, J. G. et al. NAR 2019) release v95.

Research involving human participants, their data, or biological material

Policy information about studies with human participants or human data. See also policy information about sex, gender (identity/presentation), and sexual orientation and race, ethnicity and racism.

Reporting on sex and gender	Cell lines used in this study included: HeLa and K562 (female) and IMR-5 (male) cell lines. Genome-wide and tumor mutation data were retrieved from the ENCODE, GEO, NCBI and COSMIC databases and used for this study regardless of sex.
Reporting on race, ethnicity, or other socially relevant groupings	Genome-wide and tumor mutation data were retrieved from the ENCODE, GEO, NCBI and COSMIC databases and used for this study regardless of race, ethnicity or any other socially relevant grouping.
Population characteristics	Experimental research was performed on HeLa (female) cells. ChIP-seq, OK-seq and DRIPc-seq genome-wide data were obtained from K562 (female) cell line experiments, except H3S10pho ChIP-seq data which were obtained from IMR-5 (male) cells. Tumor mutation data were retrieved from the COSMIC database, which collects data from multiple external sources. For details on the population characteristics, check COSMIC website (https://cancer.sanger.ac.uk/cosmic) and/or original publications (Tate, J. G. et al. NAR 2019).
Recruitment	Experimental research was performed on HeLa (female) cells. ChIP-seq, OK-seq and DRIPc-seq genome-wide data were obtained from K562 (female) cell line experiments, except H3S10pho ChIP-seq data which were obtained from IMR-5 (male) cells. Tumor mutation data were retrieved from the COSMIC database, which collects data from multiple external sources. For specific details on the recruitment procedure, check COSMIC website (https://cancer.sanger.ac.uk/cosmic) and/or original publications.
Ethics oversight	Experimental research was performed on HeLa (female) cells. ChIP-seq, OK-seq and DRIPc-seq genome-wide data were obtained from K562 (female) cell line experiments, except H3S10pho ChIP-seq data which were obtained from IMR-5 (male) cells. Tumor mutation data were retrieved from the COSMIC database, which collects data from multiple external sources. For specific details on the ethics oversight, check COSMIC website (https://cancer.sanger.ac.uk/cosmic) and/or original publications.

Note that full information on the approval of the study protocol must also be provided in the manuscript.

Field-specific reporting

Please select the one below that is the best fit for your research. If you are not sure, read the appropriate sections before making your selection.

☒ Life sciences ☐ Behavioural & social sciences ☐ Ecological, evolutionary & environmental sciences

For a reference copy of the document with all sections, see nature.com/documents/nr-reporting-summary-flat.pdf

Life sciences study design

All studies must disclose on these points even when the disclosure is negative.

Sample size	Sample sizes were always chosen to be large enough for each condition and biological replicate in our experiments to minimize stochastic effects and ensure reproducibility of the data. At least 50 cells per condition and biological replicate were analyzed in IF experiments.
Data exclusions	No data were excluded.
Replication	All the experiments were reliably reproduced. The experimental data results presented in this manuscript were always obtained from a minimum of three independent biological replicates. Genome-wide data retrieved from the ENCODE database were obtained from ≥2 biological replicates, except particular cases where >1 replicate data was not available. H3S10pho and FANCD2 ChIP-seq data included 2 biological replicates.<br>Cancer mutation data obtained from the COSMIC database included 400,690 tumors and 46,053,401 mutations. Tumor samples with at least 1,000 mutations (9,046 tumors with 23,013,925 SNVs and 1,636,758 indels) were used to calculate R-loop mutation burden.
Randomization	Cell samples were always chosen randomly. Pictures from microscopy slides were always taken randomly to ensure data acquisition was representative and avoid any possible bias.
Blinding	No blinding was performed as blinding was not relevant for the study since data collection and analysis were authomatically performed using different softwares ensuring unbiased results.

Reporting for specific materials, systems and methods

We require information from authors about some types of materials, experimental systems and methods used in many studies. Here, indicate whether each material, system or method listed is relevant to your study. If you are not sure if a list item applies to your research, read the appropriate section before selecting a response.

Materials & experimental systems

n/a	Involved in the study	
☐	☒	Antibodies
☐	☒	Eukaryotic cell lines
☒	☐	Palaeontology and archaeology
☒	☐	Animals and other organisms
☒	☐	Clinical data
☒	☐	Dual use research of concern
☒	☐	Plants

Methods

n/a	Involved in the study	
☒	☐	ChIP-seq
☒	☐	Flow cytometry
☒	☐	MRI-based neuroimaging

Antibodies

Antibodies used	IgG1 mouse monoclonal FANCD2 Antibody (FI17), Santa Cruz Biotechnology, Cat#sc-20022 (RRID:AB_2278211)<br>Rabbit polyclonal anti-gamma H2A.X (phospho S139) antibody, Abcam, Cat#ab2893 (RRID:AB_303388)<br>Mouse monoclonal S9.6 Antibody hybridoma HB-8730 N/A (RRID:CVCL_G144)<br>IgG2a mouse monoclonal PCNA Antibody (PC10), Santa Cruz Biotechnology, Cat#sc-56 (RRID:AB_628110)<br>Rabbit polyclonal anti-Phospho RNA polymerase II (S2) Antibody, Merck, Cat#PLA0128 (Bethyl Cat#A300-654A, RRID:AB_519341):<br>Rabbit polyclonal anti-Nucleolin Antibody, Abcam, Cat#ab50279 (RRID:AB_881762)<br>Mouse monoclonal anti-Vinculin antibody, Merck, Cat#V9264 (RRID:AB_10603627)<br>Rabbit polyclonal anti-INO80 Antibody, Abcam, Cat#ab105451 (RRID:AB_10976489)<br>Rabbit polyclonal anti-SMARCA5, Abcam, Cat#ab72499 (RRID:AB_1270821)<br>Rabbit polyclonal anti-MTA2, Sigma, Cat#HPA006214 (RRID:AB_1079421)<br>Rabbit polyclonal anti-RNaseH1, Proteintech, Cat#15606 (RRID:AB_2238624)<br>Rabbit polyclonal anti-GFP, Abcam, Cat#ab290 (RRID:AB_303395)<br>Chicken anti-Mouse IgG (H+L) Cross-Adsorbed Secondary Antibody, Alexa Fluor 594, Thermo Fisher Scientific, Cat#A21201<br>Goat anti-Mouse IgG (H+L) Highly Cross-Adsorbed Secondary Antibody, Alexa Fluor 488, Thermo Fisher Scientific, Cat#A11029<br>Goat anti-Rabbit IgG (H+L) Cross-Adsorbed Secondary Antibody, Alexa Fluor 568, Thermo Fisher Scientific, Cat#A11011

	Duolink in situ PLA probe anti-rabbit PLUS, Merck, Cat#DUO92002<br>Duolink in situ PLA probe anti-mouse MINUS, Merck, Cat#DUO92004
Validation	IgG1 mouse monoclonal FANCD2 Antibody (FI17), Santa Cruz Biotechnology, Cat#sc-20022 (RRID:AB_2278211):<br>Perez-Calero, C. et al. UAP56/DDX39B is a major cotranscriptional RNA–DNA helicase that unwinds harmful R loops genome-wide. Genes&Dev 34,1–15 (2020).<br><br>Rabbit polyclonal anti-gamma H2A.X (phospho S139) antibody, Abcam, Cat#ab2893 (RRID:AB_303388):<br>Wakita M et al. A BET family protein degrader provokes senolysis by targeting NHEJ and autophagy in senescent cells. Nat Commun 11:1935 (2020).<br><br>Mouse monoclonal S9.6 Antibody, hybridoma HB-8730 N/A (RRID:CVCL_G144):<br>Perez-Calero, C. et al. UAP56/DDX39B is a major cotranscriptional RNA–DNA helicase that unwinds harmful R loops genome-wide. Genes&Dev 34,1–15 (2020).<br>Salas-Armenteros, I. et al. Human THO–Sin3A interaction reveals new mechanisms to prevent R-loops that cause genome instability. EMBO J 36, 3532-3547 (2017).<br><br>Rabbit polyclonal anti-Nucleolin antibody, Abcam, Cat#ab50279 (RRID: AB881762)<br>Perez-Calero, C. et al. UAP56/DDX39B is a major cotranscriptional RNA–DNA helicase that unwinds harmful R loops genome-wide. Genes&Dev 34,1–15 (2020).<br><br>Mouse monoclonal anti-Vinculin antibody, Merck, Cat#V9264 (RRID:AB_10603627)<br>Perez-Calero, C. et al. UAP56/DDX39B is a major cotranscriptional RNA–DNA helicase that unwinds harmful R loops genome-wide. Genes&Dev 34,1–15 (2020).<br><br>Rabbit polyclonal anti-Phospho RNA polymerase II (S2) Antibody, Merck, Cat#PLA0128 (Bethyl Cat#A300-654A, RRID:AB_519341):<br>Stephanie J. Leuenroth and Craig M. Crews. Triptolide-induced Transcriptional Arrest Is Associated with Changes in Nuclear Substrcuture. Cancer Res 68: (13) (2018).<br><br>IgG2a mouse monoclonal PCNA Antibody (PC10), Santa Cruz Biotechnology, Cat#sc-56 (RRID:AB_628110)<br>Yang, Y. et al. DNA repair factor RAD18 and DNA polymerase PolK confer tolerance of oncogenic DNA replication stress. The Journal of Cell Biology 216(10):3097-3115 (2017).<br><br>Rabbit polyclonal anti-INO80 Antibody, Abcam, Cat#ab105451 (RRID:AB_10976489)<br>Antibody suitable for ICC/IF, IHC-Fr and WB according to Abcam website.<br>Rhee S et al. Endothelial deletion of Ino80 disrupts coronary angiogenesis and causes congenital heart disease. Nat Commun 9:368 (2018).<br><br>Rabbit polyclonal anti-SMARCA5, Abcam, Cat#ab72499 (RRID:AB_1270821)<br>Antibody suitable for ICC/IF, IHC-P, WB, IP and IHC-FrFl according to Abcam website.<br>Morris SA et al. Overlapping chromatin-remodeling systems collaborate genome wide at dynamic chromatin transitions. Nat Struct Mol Biol 21:73-81 (2014).<br><br>Rabbit polyclonal anti-MTA2, Sigma, Cat#HPA006214 (RRID:AB_1079421)<br>Antibody suitable for IF and WB according to Merck's website.<br>Used in The Human Protein Atlas.<br><br>Rabbit polyclonal anti-RNaseH1, ThermoFisher/Proteintech, Cat#15606 (RRID:AB_2238624)<br>Antibody suitable for ICC/IF, IP and WB according to ThermoFisher's website.<br>Tumini, E. et al. The Antitumor Drugs Trabectedin and Lurbinectedin Induce Transcription-Dependent Replication Stress and Genome Instability. Mol Cancer Res 17(3):773-782 (2019).<br><br>Rabbit polyclonal anti-GFP, Abcam, Cat#ab290 (RRID:AB_303395)<br>Antibody suitable for ELISA, IHC-Fr, ICC, IHC-P, IP, WB, IHC-FoFr, IHC-FrFl and Electron Microscopy according to Abcam website.<br>Chen J et al. The B-type response regulator GmRR11d mediates systemic inhibition of symbiotic nodulation. Nat Commun 13:7661 (2022).

Eukaryotic cell lines

Policy information about cell lines and Sex and Gender in Research

Cell line source(s)	HeLa cells, Homo sapiens, RRID:CVCL_0030. Obtained from The American Type Culture Collection (ATCC).
Authentication	Authentication certificate guarantee from ATCC was provided with cells upon arrival. ATCC authenticates cell lines routinely with the following tests: Short Tandem repeat (STR) Profiling, Cellular Morphology, Karyotyping, Cytochrome C Oxidase I (COI) Asaay Testing.
Mycoplasma contamination	Cell lines were tested negative for Mycoplasma contamination.
Commonly misidentified lines<br>(See ICLAC register)	No commonly misidentified cell lines were used in the study.
