# Supplementary material for: The chromatin network helps prevent cancer-associated mutagenesis at transcription-replication conflicts
Source: Nat Commun. 2023 Oct 28;14:6890. doi: 10.1038/s41467-023-42653-0 (PMC10613258; doi:10.1038/s41467-023-42653-0)
Supplement: Supplementary file 4 — Description of Additional Supplementary Files [file 41467_2023_42653_MOESM4_ESM.pdf]

Description of Additional Supplementary Files

File Name: Supplementary Data
Description: File with the accession numbers of the ENCODE ChIP-seq data used in this study
